# Study protocol: Effect of inulin supplementation on gut microbiota in patients with oral lichen planus — A double-blind, randomised, placebo-controlled parallel-group trial

**DOI:** 10.1371/journal.pone.0356180

**Published:** 2026-08-13

**Authors:** Koki Nambu, Naoki Kaneko, Shiho Yokomizo, Hu Chen, Lijing Yan, Wang Shiyao, Ryusei Shizuma, Eiji Iwata, Yukiko Ohyama, Shohei Akagawa, Soichiro Ibaragi, Shintaro Kawano, Masafumi Moriyama

**Affiliations:** 1 Section of Oral and Maxillofacial Surgery, Division of Maxillofacial Diagnostic and Surgical Sciences, Faculty of Dental Science, Kyushu University, Fukuoka, Japan; 2 OBT Research Center, Faculty of Dental Science, Kyushu University, Fukuoka, Japan; 3 Section of Oral and Maxillofacial Oncology, Division of Maxillofacial Diagnostic and Surgical Sciences, Faculty of Dental Science, Kyushu University, Fukuoka, Japan; 4 Department of Oral and Maxillofacial Surgery, Graduate School of Medicine, Dentistry and Pharmaceutical Sciences, Okayama University, Okayama, Japan; 5 Department of Pediatrics, Kansai Medical University, Osaka, Japan; Shanghai Jiao Tong University, CHINA

## Abstract

Oral lichen planus is a chronic inflammatory disease of the oral mucosa characterised by immune dysregulation, but its pathogenesis remains incompletely understood and no curative treatment has been established. Our previous work showed that patients with oral lichen planus exhibit gut dysbiosis, including reduced microbial diversity and depletion of butyrate-producing bacteria. Because butyrate promotes regulatory T-cell differentiation and supports immune homeostasis, targeting this dysbiosis may represent a microbiota-directed approach for immune modulation in oral lichen planus. This study aims to investigate the effects of inulin supplementation on the gut microbiota and related immune markers in patients with oral lichen planus. This multicentre, double-blind, randomised, placebo-controlled parallel-group trial will enrol 80 patients with oral lichen planus. Participants will be randomly assigned in a 1:1 ratio to receive either inulin (8 g/day) or a maltose placebo for 4 weeks, in addition to standard care. The primary outcome is the change in the relative abundance of prespecified butyrate-producing gut bacteria from baseline to the end of intervention at Week 6. Secondary outcomes include changes in gut microbiota diversity, salivary microbiota composition, faecal short-chain fatty acid concentrations, peripheral blood regulatory T-cell counts, blood test parameters, and clinical symptoms of oral lichen planus. Analyses will follow the intention-to-treat principle, and between-group differences will be assessed using appropriate statistical methods. This trial is designed to evaluate, in a randomised placebo-controlled setting, whether a microbiota-directed intervention can modify gut microbial profiles and related immune markers in patients with oral lichen planus. The findings are expected to clarify whether inulin supplementation can modify gut microbial profiles and related immunological markers in patients with oral lichen planus. Trial registration: UMIN Clinical Trials Registry (UMIN-CTR), UMIN000060840, registered on 1 April 2026 (https://rctportal.mhlw.go.jp/detail/um?trial_id=UMIN000060840#).

## Introduction

Oral lichen planus (OLP) is a chronic inflammatory disease of the oral mucosa characterised by T-cell–mediated immune dysregulation [1,2]. Although OLP is relatively common in daily clinical practice, its aetiology and pathogenic mechanisms remain incompletely understood, and no curative treatment has been established [3]. Histopathologically, dense T-cell infiltration, including CD4⁺ and CD8^+^ T cells is observed in the lesional mucosa, suggesting that excessive immune activation contributes to the persistent inflammation.

In recent years, the gut microbiota has been recognised as a key regulator of systemic immune function [4]. Dysbiosis has been implicated in the development of various conditions, including allergic diseases, autoimmune disorders, and inflammatory bowel disease [5]. Our previous work demonstrated that patients with OLP exhibit reduced gut microbial diversity and a marked depletion of butyrate‑producing bacteria compared with healthy individuals [6]. Because butyrate‑producing bacteria promote the differentiation and function of regulatory T cells (Tregs) [7,8], their reduction may contribute to impaired immunoregulatory capacity and the chronicity of OLP.

Inulin is a soluble dietary fibre that acts as a prebiotic and promotes the growth of beneficial gut bacteria, particularly short‑chain fatty acid (SCFA) producers such as butyrate‑, acetate‑, and propionate‑producing taxa [9,10]. Randomised controlled trials in healthy adults have shown that inulin supplementation not only alters gut microbiota composition but also increases faecal SCFA concentrations [11]. Given that OLP is characterised by dysbiosis with reduced butyrate‑producing bacteria, inulin supplementation may help restore microbial balance and modulate immune responses.

However, no studies have investigated whether microbiota‑directed interventions can influence the disease biology of OLP. Therefore, this study aims to determine whether inulin supplementation increases the abundance of butyrate‑producing gut bacteria in patients with OLP. The findings of this trial are expected to provide foundational evidence for prebiotic supplementation as an adjunctive microbiota-directed strategy in OLP.

## Materials and methods

### Study design

This study aims to determine whether inulin, a prebiotic dietary fibre, increases the abundance of butyrate-producing gut bacteria in patients with oral lichen planus (OLP). It is a multicentre, double-blind, placebo-controlled, parallel-group randomised controlled trial. Eligible participants will be randomly assigned in a 1:1 ratio to receive either inulin (8 g/day) or a maltose placebo for 4 weeks, in addition to standard care. All participants will be followed for a total of 10 weeks, consisting of a 2-week pre‑observation period, a 4-week intervention period, and a 4-week post‑observation period. Baseline assessments will be performed at Week 2, immediately before the start of the 4-week intervention, and the primary endpoint will be assessed at Week 6, corresponding to the end of the intervention. Week 10 assessments will be performed as post-intervention follow-up.

### Study setting

The trial will be conducted at Kyushu University Hospital and the Department of Oral and Maxillofacial Surgery, Okayama University Hospital. Screening, randomisation, provision of study products, and follow‑up assessments will all be performed during routine outpatient visits.

### Trial organisation and oversight

Kyushu University serves as the coordinating site and is responsible for overall trial coordination and data management. Okayama University Hospital participates as a collaborating recruitment site and is responsible for study procedures conducted at that site. No steering committee, endpoint adjudication committee, or separate data monitoring committee has been established because this is a small, low-risk investigator-initiated trial of a dietary supplement.

### Eligibility criteria

#### Inclusion criteria.

- Patients with oral lichen planus clinically diagnosed by a dentist and histopathologically confirmed.
- Men or women aged ≥18 years
- Individuals who have received a full explanation of the study and have provided written informed consent of their own free will

#### Exclusion criteria.

- Use of antibiotics within the past 3 months
- Hospitalisation within the past 3 months
- Refusal to participate in the study
- Pregnant or possibly pregnant individuals
- Known food allergy to inulin or plants of the Asteraceae family
- Any other condition deemed inappropriate for participation by the principal investigator

### Recruitment and consent

Potential participants will be identified during routine outpatient care. Eligible individuals will receive written study information and an oral explanation from study staff, after which written informed consent will be obtained. Participation is voluntary, and participants may withdraw at any time without affecting their clinical care.

### Randomization

A stratified permuted block randomisation sequence will be generated before the start of enrolment by an independent allocation manager who is not involved in participant recruitment, treatment, or outcome assessment. Randomisation will be stratified by study site (Kyushu University Hospital and Okayama University Hospital) using variable block sizes to minimise the predictability of allocation. The allocation table for 80 participants will be generated using computer-based randomisation software and stored securely by the allocation manager.

After written informed consent is obtained and eligibility is confirmed, the enrolling investigator will contact the allocation manager to request the treatment assignment for the participant. The allocation manager will assign the participant to the next available allocation in the pre-generated sequence. At the time of each allocation, the allocation manager will record the registration date, date of consent, participant identification code, and the name of the enrolling investigator in a separate allocation log. The enrolling investigator will have no access to the allocation table or to future assignments, thereby ensuring adequate allocation concealment.

### Blinding

A blinding officer, independent of the allocation manager and enrolling investigators, will be responsible for ensuring the indistinguishability of the study products. The blinding officer will prepare a correspondence table linking sequential product serial numbers, the randomisation order, and product allocation codes (inulin or maltose). Based on this table, the blinding officer will label all study product sachets with their respective allocation codes only, so that neither participants nor investigators can identify the contents.

The correspondence table will be kept securely by the blinding officer and will not be disclosed to any other study personnel until unblinding. Unblinding will occur only in the event of a medical emergency requiring knowledge of the allocated treatment for appropriate clinical management of the individual participant. Full unblinding of all participants will be performed only after the database is locked and the analysis population and case eligibility decisions have been finalised.

Participants, enrolling investigators, outcome assessors, and data analysts will remain blinded throughout the study. During data analysis, group assignments will be coded (e.g., Group A and Group B) so that analysts remain unaware of which group received inulin and which received placebo. Unblinding of the data analysts will occur only after the primary analysis has been completed and the results have been locked.

### Interventions

#### Inulin group.

Participants in the inulin group will consume Inulia® IQ (inulin, 4 g per sachet), taken twice daily (8 g/day total) for 4 weeks.

#### Placebo group.

Participants in the placebo group will consume maltose powder identical in appearance (4 g per sachet), taken twice daily (8 g/day total) for 4 weeks.

#### Product matching.

Both the inulin and placebo products are white, odourless powders supplied in identical sachets labelled only with allocation codes. Both products are matched in appearance, texture, packaging, and dissolve readily in water with a mildly sweet taste, minimising the likelihood of unblinding.

### Concomitant treatments

Standard treatment for OLP, including topical corticosteroids, may be continued during the study period. Participants will be instructed to maintain their usual treatment regimen whenever clinically feasible. Any initiation, discontinuation, or dose modification of OLP-related treatments during the study period will be recorded. No dietary restrictions will be imposed, but participants will be instructed to maintain their usual dietary habits and to avoid initiating new probiotic, prebiotic, or fibre supplements other than the assigned study product. The use of inulin-containing supplements will be prohibited. Adherence will be monitored using the daily dietary record, in which participants will record whether they consumed the assigned study product each day.

### Participant timeline

**Fig 1:**
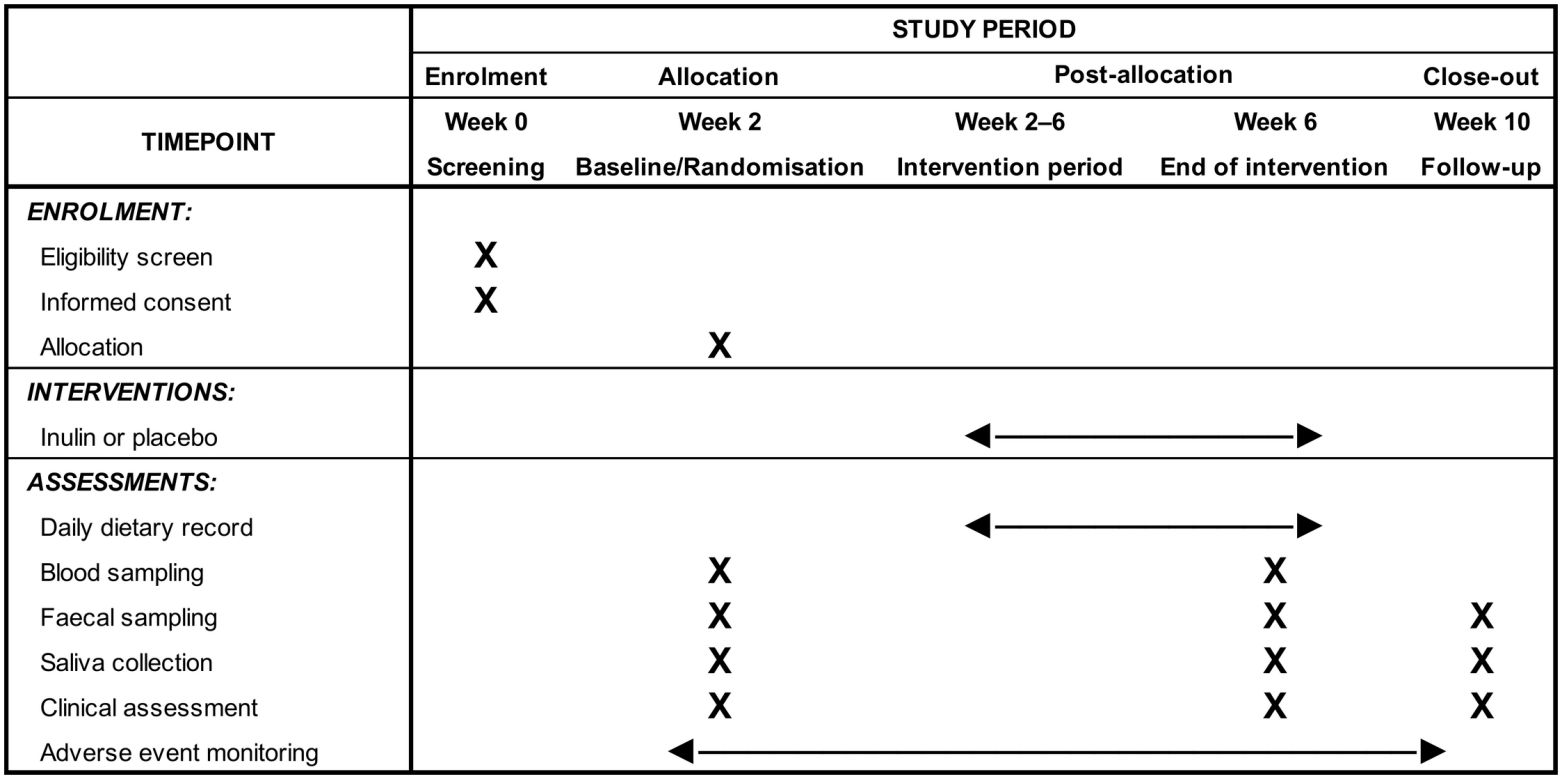
SPIRIT schedule of enrolment, allocation, interventions, and assessments.

The schedule of enrolment, allocation, interventions, and assessments is shown in Fig 1.

This figure summarises the timing of participant enrolment, randomisation, intervention, dietary assessment, biospecimen collection, clinical assessments, and adverse event monitoring during the trial.

### Outcomes

#### Primary outcome.

The primary outcome is the change from baseline to Week 6 in the summed relative abundance of prespecified butyrate‑producing taxa, including *Faecalibacterium prausnitzii*, *Anaerostipes hadrus*, the *Eubacterium hallii* group (including *Anaerobutyricum* spp.), *Agathobacter rectalis*, and *Fusicatenibacter saccharivorans*. These taxa were selected based on their established role in colonic butyrate production and their depletion in patients with OLP in our previous study [6].

#### Secondary outcomes.

Secondary outcomes include changes in gut and salivary microbiota composition and diversity, faecal short-chain fatty acid concentrations, peripheral blood Treg counts, blood test parameters (white blood cell count, eosinophil count, and serum IgE), and clinical symptoms of OLP. Clinical severity will be assessed using the Oral Lichen Planus Disease Activity Score (OLP-DAS) [12]. These outcomes are intended to capture the broader biological and clinical effects of inulin supplementation beyond the prespecified primary taxa.

#### Exploratory outcomes.

Exploratory outcomes will include additional analyses aimed at generating mechanistic insights into the effects of inulin supplementation. These will comprise changes in other butyrate‑producing taxa not included in the primary outcome, extended profiling of faecal short‑chain fatty acids, exploratory correlations between gut microbiota composition and peripheral blood Treg counts, phenotypic characterisation of Treg subsets, and post‑hoc responder versus non‑responder analyses. Dietary assessment during the intervention period will be conducted using a daily dietary record to monitor short-term changes in eating behaviour that may influence microbiota or clinical responses. A copy of the daily dietary record is provided as S3 File. Details of these outcomes, including assessment methods and time points, are summarised in Table 1.

**Table 1:** Primary, secondary, and exploratory outcomes, including assessment methods and time points.

Outcome category	Outcome	Method	Time points
Primary	Change in the summed relative abundance of prespecified butyrate-producing taxa*	16S rRNA gene sequencing; QIIME 2 pipeline	Weeks 2 (baseline), 6 (end of intervention), and 10 (follow-up)
Secondary	Gut microbiota composition	16S rRNA gene sequencing of faecal samples; QIIME 2 pipeline	Weeks 2, 6, and 10
	Gut microbiota diversity	Alpha-diversity (Shannon, Simpson, Faith's PD) and beta-diversity (Bray-Curtis, UniFrac)	Weeks 2, 6, and 10
	Faecal organic acid concentrations (SCFAs)	HPLC-MS/MS or GC for butyrate, acetate, and propionate	Weeks 2 and 6
	Salivary microbiota composition	16S rRNA sequencing of saliva samples; QIIME 2 pipeline	Weeks 2, 6, and 10
	Blood test parameters	Automated haematology and biochemistry analyser	Weeks 2 and 6
	Peripheral blood Treg counts	Flow cytometry (CD4 + CD25 + Foxp3+); standardised gating strategy (lymphocytes - > singlets - > CD3 + CD4 + - > CD25 + Foxp3+); central analysis at Kyushu University	Weeks 2 and 6
	Clinical symptoms of OLP	Oral Lichen Planus Disease Activity Score (OLP-DAS)	Weeks 2, 6, and 10
Exploratory	Additional butyrate-producing taxa	16S rRNA sequencing; genus- and species-level profiling	Weeks 2, 6, and 10
	Extended SCFA profiling	HPLC-MS/MS or gas chromatography	Weeks 2 and 6
	Microbiome-immune correlations	Integrated analysis of microbiota and Treg data	Weeks 2 and 6
	Treg phenotypic subsets	Extended flow cytometry markers (e.g., CD45RA + /-, Helios + /-)	Weeks 2 and 6
	Responder versus non-responder analysis	Unsupervised clustering integrating microbiome, immune, and clinical parameters	Post hoc
	Dietary intake during intervention	Daily dietary record	Week 2–6

*Prespecified taxa include *Faecalibacterium prausnitzii*, *Anaerostipes hadrus*, the *Eubacterium hallii* group (including *Anaerobutyricum* spp.), *Agathobacter rectalis*, and *Fusicatenibacter saccharivorans*.

### Sample size calculation

In our previous cross-sectional study, the mean relative abundance of butyrate-producing bacteria was approximately 12.4% (SD 8.1%) in patients with OLP and 14.7% (SD 8.2%) in healthy controls. Prior randomised trials in healthy adults have reported that inulin or inulin-type fructan supplementation at comparable doses can increase the relative abundance of fermentative taxa by approximately 5–15 percentage points, with concurrent increases in faecal short-chain fatty acid concentrations. On the basis of these data, we assumed that inulin supplementation would increase the summed relative abundance of the five prespecified butyrate-producing taxa by 12.5 percentage points from baseline, compared with 2.5 percentage points in the placebo group, yielding an expected between-group difference of 10 percentage points.

Because no prior intervention study has examined this response in patients with OLP, a conservative common standard deviation of 15% was adopted to account for expected variability in this patient population. Based on a two-sample comparison of the primary outcome between groups, assuming a two-sided significance level of 0.05, 80% power, and a standardised effect size of approximately 0.67, approximately 36 participants per group are required. Allowing for approximately 10% dropout, the target sample size was set at 40 participants per group, for a total of 80 participants. The calculation was performed using G*Power version 3.1.9.

### Statistical analysis

All analyses will follow the intention-to-treat principle. Statistical tests will be two-sided with a significance level of 0.05 unless otherwise specified. Analyses will be performed using R (version 4.3.1 or later).

The primary outcome is the change from baseline to Week 6 in the combined relative abundance of five prespecified butyrate-producing taxa. The between-group difference will be assessed using a two-sample t-test or Mann–Whitney U test depending on distributional assumptions. ANCOVA adjusting for baseline values will be performed as a prespecified supplementary analysis. Because 16S rRNA gene sequencing data are compositional, a sensitivity analysis of the primary outcome using centred log-ratio transformation will be performed.

Secondary continuous outcomes will be analysed using ANCOVA models adjusting for baseline values. Repeated measures will be analysed using linear mixed-effects models with random intercepts to account for within-participant correlations and to evaluate time-by-group interactions.

Microbiome sequencing data will be processed using the QIIME 2 [13,14] platform. Taxonomic assignment will be performed using a prespecified reference database and classifier. Because species-level classification by 16S rRNA gene sequencing may be limited for some taxa, the primary analysis will use the most resolved taxonomic level that can be reliably assigned by the sequencing region and classifier. Taxa corresponding to the prespecified butyrate-producing bacteria will be aggregated according to a predefined taxonomic mapping before unblinding. Alpha diversity indices will be compared using parametric or non-parametric tests as appropriate. Beta diversity will be analysed using PERMANOVA based on UniFrac and Bray–Curtis distance matrices. Differential abundance analysis will be performed using LEfSe [15], with ANCOM-BC and/or ALDEx2 used as complementary approaches to account for the compositional nature of microbiome data. For multiple related comparisons, particularly microbiome taxa analyses, short-chain fatty acid comparisons, and immunological marker panels, false discovery rate correction using the Benjamini-Hochberg procedure will be applied. Adjusted p values will be reported alongside unadjusted p values where applicable.

Exploratory analyses, including changes in individual taxa, microbiome-immune correlations, Treg phenotypic subset analyses, and responder versus non-responder comparisons, will be considered hypothesis generating. Dietary intake data from the daily dietary record will be summarised descriptively.

The primary analysis will include all randomised participants with available baseline and Week 6 primary outcome data, according to the intention-to-treat principle. Missing outcome data will be examined for patterns and likely mechanisms. If missingness is judged to be at random, multiple imputation using chained equations will be performed as a sensitivity analysis. Adverse events will be summarised descriptively by treatment group. No interim analyses are planned.

### Safety monitoring

Adverse events (AEs) will be recorded throughout the study period. An AE is defined as any unfavourable or unintended disease, sign, symptom, or abnormal laboratory finding occurring in a participant, regardless of its causal relationship with the study. Expected AEs related to the study products include diarrhoea, soft stools, abdominal bloating, and food allergy; the risk of these events is considered low. Blood samples of 40 mL before the intervention and 60 mL immediately after the intervention will be collected for immunological analyses, and the risk of anaemia is considered very low. If an AE occurs, the investigators will provide appropriate medical care and record the event in the medical records. The study product may be discontinued if necessary. Participants may be withdrawn if they request withdrawal, become ineligible after enrolment, experience an AE or worsening of symptoms that makes continuation inappropriate, substantially deviate from the protocol, fail to comply with the intervention, or if the principal investigator judges that continuation is difficult. Participants who discontinue the study product will be asked to continue scheduled follow-up assessments where possible.

Trial continuation will be reviewed if important safety or efficacy information becomes available, recruitment is judged to be extremely difficult, the study objective is achieved before the planned sample size or study period is reached, or protocol changes requested by the ethics committee cannot be implemented. If the ethics committee recommends or instructs termination, or if the principal investigator decides to suspend or terminate the study, the reason will be promptly reported to the ethics committee and the head of the study institution.

In the event of study-related harm, medical care will be provided through the usual health insurance system.

### Data management

Data will be managed using participant identification codes. Documents will be stored for 10 years, and biological samples for 5 years. Specific consent will be obtained from participants for the potential future use of these stored specimens and de-identified data in ancillary studies. Participants may opt out of this future use without any penalty.

Personal information will be strictly protected. Identifiable data will be removed and replaced with study‑specific identification codes. The correspondence table linking identification codes with personal information will be securely stored and accessible only to authorised study personnel. Study documents will be retained at Kyushu University for the required period, and biological samples will be stored in locked facilities and disposed of according to institutional procedures after the retention period.

### Data sharing and dissemination

This article does not report any datasets generated or analysed during the current study protocol stage. After completion of the trial and publication of the primary trial results, raw 16S rRNA gene sequencing data will be deposited in the DDBJ Sequence Read Archive. De-identified participant-level data, processed microbiome data, metadata, and a data dictionary underlying the completed trial findings will be made available as Supporting Information files. Accession numbers, DOIs, or other persistent identifiers will be provided in the primary results publication. Data sharing will be conducted in accordance with the ethics-approved protocol, participant consent, and institutional regulations.

### Ethical considerations

This study was approved by the Clinical Research Ethics Committee of Kyushu University Hospital (approval number: 25014; initial approval date: 6 February 2026; amendment approval date: 16 July 2026). Any substantial modifications to the protocol, such as changes to eligibility criteria, outcomes, or statistical analyses, will be submitted to the same committee for review and approval prior to implementation. Such amendments will also be updated in the UMIN clinical trials registry (UMIN000060840). All procedures will be conducted in accordance with the Declaration of Helsinki (revised 2024) and the Ethical Guidelines for Medical and Biological Research Involving Human Subjects in Japan. Written informed consent will be obtained from all participants prior to enrolment.

### Trial status

At the time of manuscript submission (24 May 2026), this trial is ongoing. The first participant was enrolled in April 2026. Participant recruitment has begun but is not complete, data collection is not complete, and no outcome analyses have been performed.

The planned recruitment period extends from ethics committee approval to 31 March 2029, and participant recruitment is expected to be completed by 31 March 2029. Based on clinical records at the participating institutions, approximately 80 patients with OLP are seen per year across the two sites combined. After applying the eligibility criteria — particularly the exclusion of patients who have used antibiotics within the preceding 3 months — an estimated 50% (approximately 40 patients per year) are expected to be eligible. Assuming a consent rate of approximately 65–70%, we project that around 26–28 patients per year can be enrolled, and the target sample size of 80 participants is therefore expected to be achieved within approximately 3 years after the first enrolment.

Each participant will be followed for 10 weeks after enrolment. Therefore, if the final participant is enrolled by 31 March 2029, data collection for the final participant is expected to be completed by June 2029. Data cleaning and statistical analyses will begin after completion of data collection, and the primary results are expected to become available in 2029. The overall study period is planned to continue until 31 March 2031, as specified in the ethics committee-approved protocol, allowing time for trial completion procedures, dissemination, and administrative close-out. Should recruitment fall below the projected rate, strategies such as extending referral networks to affiliated hospitals or increasing awareness among local dental practitioners will be considered.

This protocol was developed in accordance with the SPIRIT (Standard Protocol Items for Randomized Trials) guidelines. This manuscript describes protocol version 1.1, dated 7 July 2026. The completed SPIRIT checklist is provided as S1 File, and the full study protocol is provided as S2 File.

## Discussion

This trial addresses an important unanswered question of whether modulation of the gut microbiota may influence the disease biology of oral lichen planus (OLP). Previous studies have shown that patients with OLP exhibit reduced gut microbial diversity, particularly depletion of butyrate-producing bacteria [6], which are involved in regulatory T-cell (Treg) differentiation [7,8]. Targeting gut dysbiosis may therefore represent a novel approach to immune modulation in OLP. This trial is designed to examine whether inulin supplementation influences these biological pathways in patients with OLP.

The study has several methodological strengths. The double-blind, placebo-controlled design is intended to minimise bias, and the simultaneous assessment of gut microbiota, salivary microbiota, faecal short-chain fatty acids, and peripheral blood Treg counts will enable an integrated evaluation of the gut-immune-oral axis [16,17].

Several limitations should be acknowledged. The sample size is powered to detect changes in the prespecified butyrate-producing taxa but may be insufficient to detect small differences in clinical symptoms. The relatively short intervention period also limits assessment of longer-term effects. Dietary habits may influence the gut microbiota [18,19]; although participants will be instructed to maintain their usual diet and dietary intake will be monitored using a daily dietary record, residual confounding by diet cannot be fully excluded. In addition, although maltose was selected as the placebo because it is rapidly absorbed in the small intestine and is not expected to reach the colon in substantial amounts, minor indirect effects on the gut microbiota cannot be completely excluded.

Overall, this trial is expected to provide a structured evaluation of the effects of inulin supplementation on the gut microbiota and related immune parameters in patients with OLP. The findings may contribute to a better understanding of the biological pathways linking gut dysbiosis and disease activity in this condition.

## Supporting information

S1 File SPIRIT checklist. (DOCX)

S2 File Full study protocol. (PDF)

S3 File Daily dietary record. (DOCX)
