# Supplementary material for: Study protocol: Effect of inulin supplementation on gut microbiota in patients with oral lichen planus — A double-blind, randomised, placebo-controlled parallel-group trial
Source: PLoS One. 2026 Aug 13;21(8):e0356180. doi: 10.1371/journal.pone.0356180 (PMC13472398; doi:10.1371/journal.pone.0356180)
Supplement: Supplementary file 1 [file pone.0356180.s002.pdf]

試験計画書

口腔扁平苔癬患者を対象とした
イヌリン摂取による腸内細菌叢の変化の検討
(プラセボ二重盲検ランダム化並行群間比較試験)

試験責任者名　森山　雅文
所属機関名　九州大学大学院歯学研究院 口腔顎顔面病態学講座 口腔顎顔面外科学分野

版番号：第1.1版
作成年月日：2026年7月7日

目次

0. 概要 3
1. 試験題目 5
2. 目的 5
3. 背景と意義 5
4. 試験対象者の選択 6
5. 試験の方法 6
6. スケジュールと試験期間 8
7. 試験実施後の対応 9
8. 評価項目 9
9. 試験対象者にとっての経済的負担あるいは謝礼等 9
10. 予期される利益と不利益 9
11. 有害事象が発生した際の対応 10
12. 試験対象者に対する健康被害発生時の対応と補償等 10
13. 個々の試験対象者における中止基準と試験中止時等の対応 10
14. 目標症例数とその設定根拠、及び統計解析方法 11
15. 倫理的事項 12
16. 個人情報等の取扱い 12
17. 試料・情報の保管及び廃棄の方法 12
18. 試料・情報の二次利用について 13
19. 試験機関の長への報告 13
20. データの収集及びデータマネジメント 13
21. 試験対象者等及びその関係者からの相談等への対応 13
22. 個々の試験対象者における試験結果の取扱い 14
23. 試験成果の発表方法 14
24. 試験者等の利益相反に関する状況 14
25. 特許権等の知的財産権について 14
26. 試験の実施体制(統計解析、委託機関等も含む) 14
27. その他:試験に関する情報公開(ホームページ) 15
28. 参考資料・文献リスト 16

0. 概要

0.1 試験題目

「口腔扁平苔癬患者を対象としたイヌリン摂取による腸内細菌叢の変化の検討」

0.2 目的

近年、腸内細菌叢の乱れ(dysbiosis)が自己免疫疾患、炎症性腸疾患、アレルギー疾患発症と関連すると報告されている[1-5]。また、私たちは口腔領域において、口腔扁平苔癬患者の腸内細菌叢は酪酸産生菌の減少に特徴づけられるdysbiosisを来たしていることを明らかにした。一方で、イヌリンは水溶性食物繊維であり、プレバイオティクスとして腸内で酪酸産生菌を増加させる。今回の試験では、口腔扁平苔癬患者にイヌリンを摂取させることで腸内細菌叢に占める酪酸産生菌が増加するか否かを明らかにすることを目的とする。

0.3 対象(適格基準・除外基準)

＜適格基準＞

1)歯科医師により口腔扁平苔癬と診断された患者
2)18 歳以上の男女
3)本試験への参加にあたり十分な説明を受けた後、本人による十分な理解の上、自由意思による文書同意が得られた者

＜除外基準＞

1)直近 3 ヶ月間以内に抗菌薬を使用した者＊
2)3 ヶ月以内に入院歴のある者
3)本人が本試験の参加について拒否した者
4)妊婦又は妊娠の可能性のある患者
5)イヌリンや原材料となるキク科植物に対する食物アレルギーを持つ者
6)その他、試験責任者が試験対象者として不適当と判断した者

＊試験中に抗菌薬を使用した者についても試験対象から除外する。

0.4 試験のデザイン

本試験に際してはプラセボ対照二重盲検ランダム化並行群間比較試験を予定している。

0.5 評価項目

＜主要評価項目＞

イヌリン摂取前後の腸内細菌叢に占める酪酸産生菌割合の変化量

＜副次評価項目＞

以下の項目の機能性イヌリン摂取前後の変化量

1. 腸内細菌叢の差異
2. 血液検査データ(白血球数、好酸球数、IgE 値、特異的 IgE 値)
3. 疾患の症状(粘膜炎の範囲、程度)
4. 腸内細菌叢、便中有機酸濃度:腸内細菌と腸内細菌が作る物質の詳細な分析結果のこと。
5. 末梢血制御性 T 細胞数:追加で採血した血液を使って解析する。
6. 唾液細菌叢の差異

0.6 目標登録症例数

口腔扁平苔癬: 80 例

0.7 試験期間

九州大学病院臨床試験倫理審査委員会の承認日より 2031 年 3 月 31 日まで

0.8 シェーマ

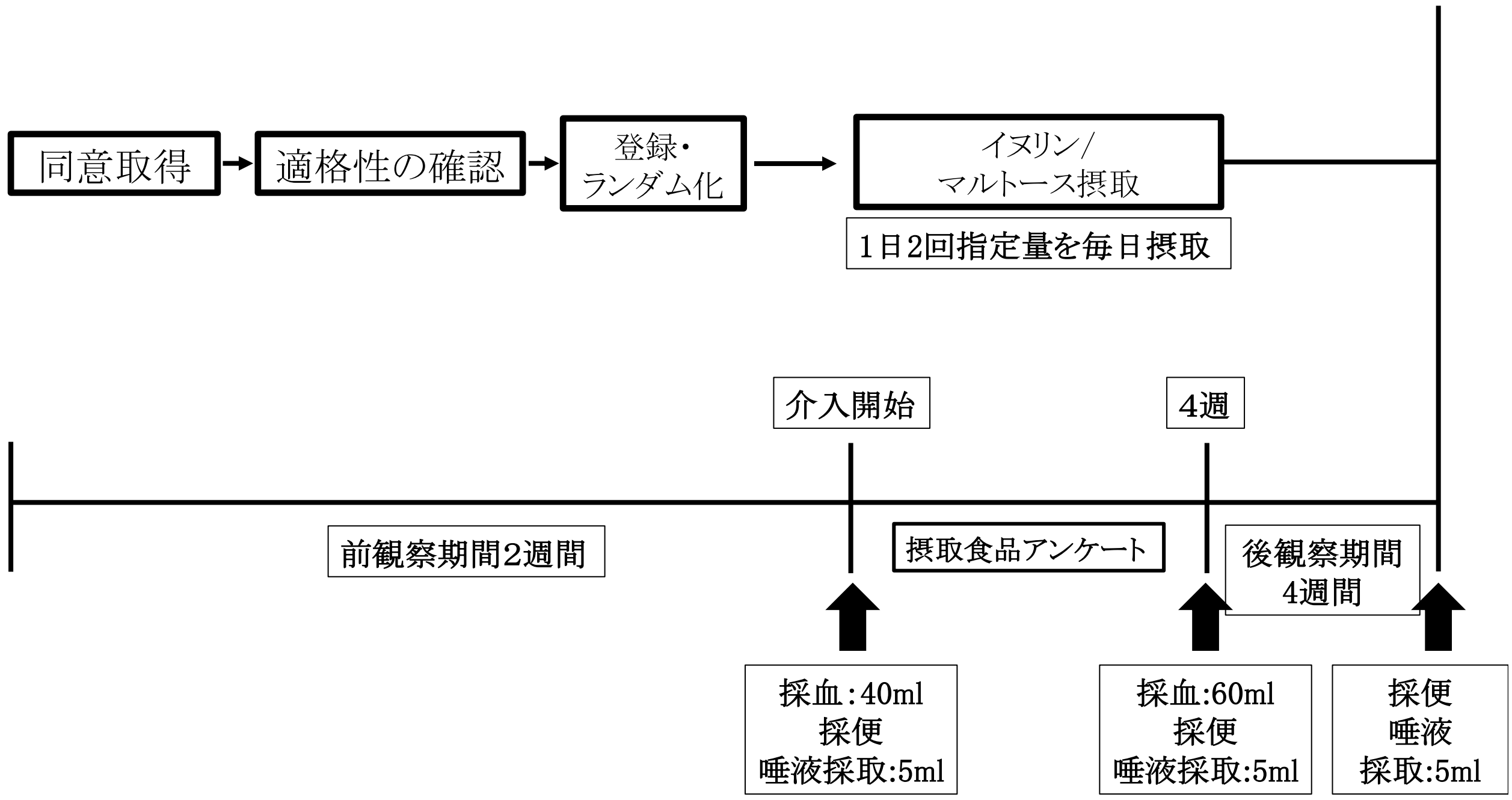

1．試験題目

「口腔扁平苔癬患者を対象としたイヌリン摂取による腸内細菌叢の変化の検討」(プラセボ対照二重盲検ランダム化並行群間比較試験)

2．目的

口腔扁平苔癬患者にイヌリンを摂取させることで腸内細菌叢に占める酪酸産生菌が増加するか否か、dysbiosis の有無を明らかにすることを目的とする。

3．背景と意義

口腔扁平苔癬は免疫系の異常により口腔粘膜に慢性炎症を引き起こす疾患あり、歯科診療において比較的高頻度で遭遇するが、病因や発症機序の多くは未解明で、根本的治療法は確立されていない。口腔扁平苔癬では病変部に CD4 陽性 T 細胞が浸潤していることが知られており、われわれもその関与を報告してきた。[1.2)]

一方で、ヒトの腸管内には1000種類以上、40兆個以上の細菌が棲みつき腸内細菌叢（gut microbiota）を形成しており、宿主であるヒトの健康に大きく関与する。近年、アレルギーや自己免疫疾患、炎症性腸疾患などでは腸内細菌叢の異常（dysbiosis）が病態に深く関与することが相次いで報告されている。[3)-6)]
我々はこれまでの研究で口腔扁平苔癬の患者の腸内細菌叢では健常者（HC）と比較して多様性が低く、特に酪酸産生菌の減少を確認した。[7)] これらの菌は腸管内で制御性 T 細胞（regulatory T cell: Treg）の誘導する働きを有し、Treg は過剰な免疫応答を抑える機能を有するため、酪酸産生菌の減少が口腔扁平苔癬病変における炎症の持続と病態の慢性化に関連する可能性がある。実際これまでの研究においても腸内細菌の減少と免疫抑制機能をもつ Treg の減弱には正の相関関係があることが認められた。[7,8)]

イヌリンは水溶性食物繊維であり、腸管内でプレバイオティクスとして作用し、腸内細菌叢を改善する。本邦で実施された健康成人 120 名に対するビフィズス菌及びイヌリン摂取のランダム化二重盲検クロスオーバー試験では、2週間の摂取によって腸内細菌叢が変化したのみならず、便中の酪酸・酢酸・プロピオン酸など有用短鎖有機酸が増加した。[10)] そこで、酪酸産生菌の減少に特徴づけられる dysbiosis を来たしていると考えられる口腔扁平苔癬患者にイヌリンを摂取させることで、腸内細菌叢に占める酪酸産生菌を増加させることができるか否かを明らかにすることを本試験の目的とする。

本試験により、イヌリンを用いて口腔扁平苔癬患者の酪酸産生菌を増加させることができることが確認できれば、根本的な治療方法の確立がなされていない同疾患の治療法の開発に発展させることが期待でき、社会的なニーズも高いと考える。

4. 試験対象者の選択

4.1. 適格基準

以下の基準をすべて満たす患者を対象とする

1. 歯科医師により口腔扁平苔癬と診断された患者
2. 18 歳以上の男女
3. 本試験への参加にあたり十分な説明を受けた後、本人による十分な理解の上、自由意思による文書同意が得られた者

4.2. 除外基準

以下のうちひとつでも該当する場合には対象から除外する。

1. 直近 3 ヶ月間以内に抗菌薬を使用した者＊
2. 3 ヶ月以内に入院歴のある者
3. 本人が本研究の参加について拒否した者
4. 妊婦又は妊娠の可能性のある患者
5. イヌリンや原材料となるキク科植物に対する食物アレルギーを持つ者
6. その他、試験責任者が試験対象者として不適当と判断した者

＊試験中に抗菌薬を使用した者についても試験対象から除外する。

5. 試験の方法

5.1. 概要

5.1.1. 試験のデザイン

プラセボ対照二重盲検ランダム化並行群間比較試験

5.1.2. 試験のアウトライン

同意が取得できた患者について、適格基準、除外基準への該当を再度確認した後、あらかじめ用意した割付表に基づきランダムにイヌリン摂取群、プラセボ摂取群に分類する。介入開始時に採血(40ml)、採便、唾液採取(5ml)を実施。両群ともに試験食品を 1 回1包(4g)1 日 2 回、計4週間摂取する。摂取期間中は摂取食品アンケートに毎日回答頂く。介入終了時に採血(60ml)、採便、唾液採取(5ml)を行う。また、介入終了 1 ヶ月後に採便、唾液採取(5ml)を実施する。

5.1.3. 試験対象者の参加予定期間

摂取期間は 4 週間 前観察期間が 2 週間、後観察期間が 4 週間

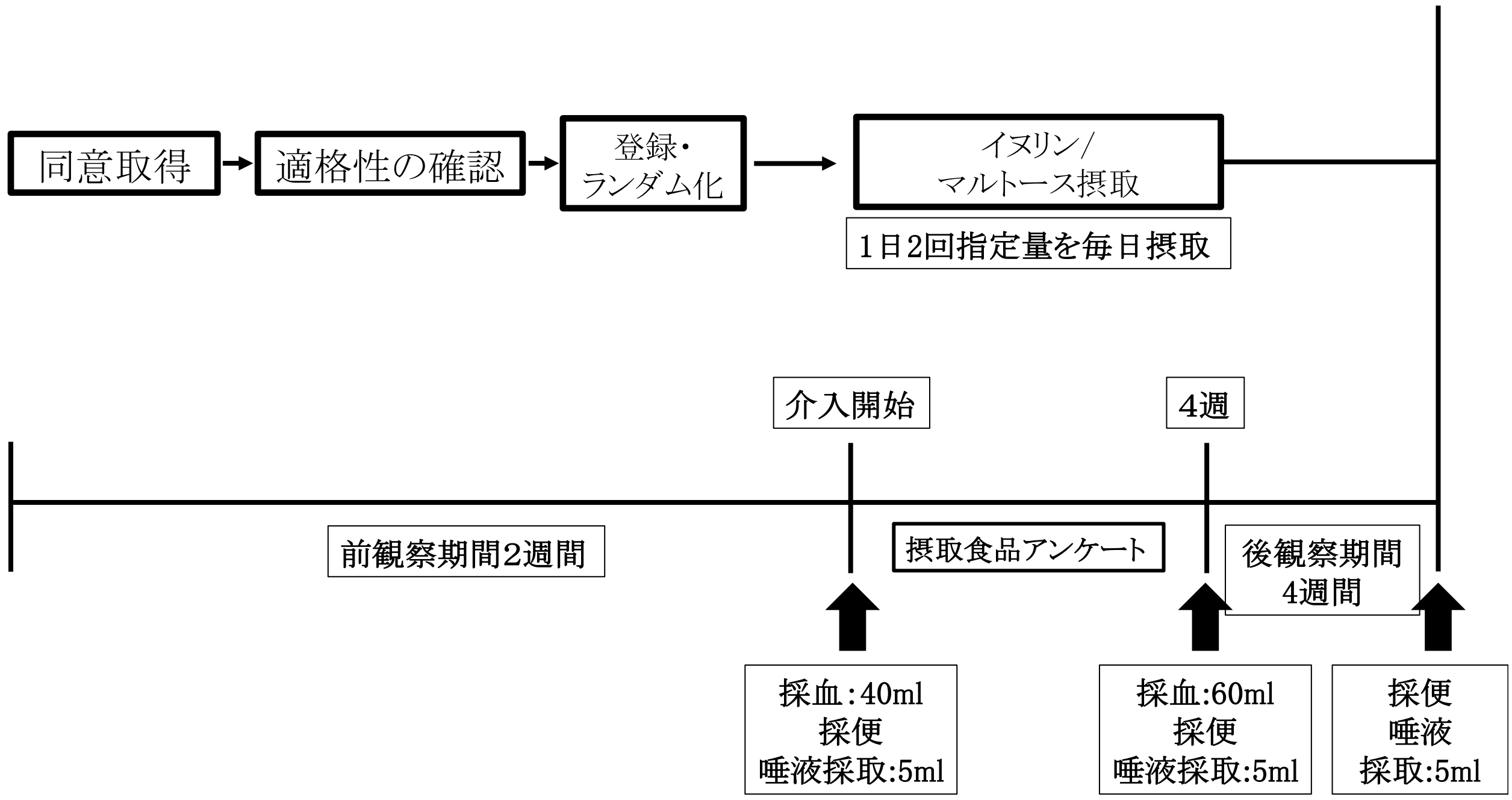

5.2. 登録の方法

試験責任者又は試験分担者(以下、試験担当者)は、試験対象者に対して試験対象者識別コードを付与し、適格性を確認の上症例登録を行う。この際試験責任者が保管する試験対象者識別コードリストに必要な事項を記載する。

5.3. 割付の方法

割付責任者はあらかじめ試験食品のランダム割付80症例分の割付表を作成し適切に保管する。試験責任者あるいは試験分担者は、試験対象者からの同意が取得されたのち速やかに割付責任者に当該試験対象者の試験治療の割付を照会する。割付責任者は、事前に作成した割付表にもとづき、若い順番ずつ割付表に記載されている試験治療を試験担当者に通知する。割付責任者は、割付の通知をした際には速やかに、「登録日付」、「同意取得日」、「登録された試験対象者の識別コード」、「試験対象者登録をした試験担当者名」を割付記録表に記入し保管する。

試験食品盲検化担当者は、試験食品の識別不能性を確認する。試験食品盲検化担当者は、通し番号、ランダムな順序のプラセボ及び試験食品（イヌリン、マルトース）、及び試験食品割付コード（記号・番号）からなる対応表（試験食品割付コード表）を作成する。試験食品盲検化担当者は、試験食品割付コード表に基づき試験食品に試験食品割付コード名を付す。試験食品割付コード表は、開鍵時まで試験食品盲検化担当者が保管し、他の者には開示しない。開鍵は、緊急避難的に該当症例について行う場合を除いて、試験が終了し各症例の採否が固定するまで行わない。

5.4. 試験食品の投与方法

5.4.1. 試験食品の概要

(1)製品名:イヌリア®IQ

原材料名:イヌリン

含量:4g/包

貯法:常温

製造者:SENSUS 社(オランダ)
輸入者:伊藤忠商事株式会社　東京都港区北青山 2-5-1
個包装:株式会社ウメケン　大阪府大阪市北区黒崎町 3-16
提供者:バブルスター株式会社　神奈川県大和市代官 1-15-15

(2)対照食品:プラセボ(サンマルト　シロ)
原材料名:マルトース
含量:4g/包
貯法:常温
製造者:ナガセヴィータ株式会社　　岡山県岡山市北区下石井 1-1-3
個包装:株式会社ウメケン　大阪府大阪市北区黒崎町 3-16
提供者:バブルスター株式会社　神奈川県大和市代官 1-15-15

5.4.2. 試験食品の投与方法

前観察期間の後、割付にしたがいイヌリンまたはプラセボを摂取する。
投与期間は摂取開始から4週間とし、1日4g×2回摂取する。
2包(8g)/day
投与量の設定根拠:これまで8gのイヌリン摂取にて腸内環境が改善されたという先行研究に従った。

5.4.3. 併用薬(療法)についての規定

タマネギやニンニクなど、イヌリンを含む一般食品に含まれるイヌリンの量は少なく、日常的な摂取による影響は極めて限定的であるため、これらの食品は制限しない。一方で、イヌリンを添加したサプリメントや強化食品は摂取量が大きく変わる可能性があるため、試験期間中は禁止とする。
試験期間中の口腔扁平苔癬に対する治療（局所ステロイド塗布）は継続可能とする。

5.4.4. 減量及び休薬についての規定

該当なし

6. スケジュールと試験期間

下記に示したスケジュールに従って以下の観察・検査・評価を実施する。

項目	前観察	投与開始	投与期間	後観察	後観察
時期	2週前	0週	4週	投与終了時	終了後4週
受診	○			○	○
同意取得	○				
患者背景の確認	○				
試験食品摂取			○		
摂取食品アンケート回答			○		
自覚症状・他覚所見	○	○		○	○
有害事象の観察		←→	←→	←→	←→
採血		○		○	
採便		○		○	○
唾液採取		○		○	○

試験期間は下記のとおりである。
登録期間：九州大学病院臨床試験倫理審査委員会の承認日より 2029 年 3 月 31 日まで
追跡期間：症例登録日より 10 週間
試験期間：承認日より 2031 年 3 月 31 日まで

7. 試験実施後の対応

該当なし

8. 評価項目

＜主要評価項目＞

イヌリン摂取前後の腸内細菌叢に占める酪酸産生菌割合の変化量

＜副次評価項目＞

以下の項目の機能性イヌリン摂取前後の変化量

1. 腸内細菌叢の差異
2. 血液検査データ（白血球数、好酸球数、IgE 値、特異的 IgE 値）
3. 疾患の症状 （粘膜炎の範囲、程度等）
4. 腸内細菌叢、便中有機酸濃度*
5. 末梢血制御性 T 細胞数：追加で採血した血液を使って解析する。
6. 唾液細菌叢の差異

＊：腸内細菌と腸内細菌が作る物質の詳細な分析結果のこと。本研究では得られた糞便サンプルを匿名化したのち、タカラバイオ株式会社に解析を委託する。

9. 試験対象者にとっての経済的負担あるいは謝礼等

本試験のうち、腸内細菌叢の解析、便中有機酸濃度測定およびTreg数測定は試験責任者が所属する診療科の研究費で賄う。それ以外は通常の保険診療内で行われるため、試験に参加することによる試験対象者の追加費用負担は発生しない。謝礼として、来院1回につき1万円相当の図書カードを渡す（介入開始時、介入終了時、介入終了1ヶ月後の計3回）。試験を途中で中止した場合でも、それまでに来院した回数分の謝礼を渡し、未実施の来院分については謝礼は発生しない。

10.予期される利益と不利益

10.1. 本試験により予期される利益

イヌリンまたはマルトースを無償で提供される。また、ランダム化により対照群となった場合でも、試験期間終了後に、希望があればイヌリン4週間分を提供する。また、試験成果により将来の医療の進歩に貢献できる可能性がある。

10.2. 本試験により予期される不利益

10.2.1 侵襲等の負担の内容

本試験は日常診療において検体の提供を受ける試験である。そのため、試験目的で行う採血、採便および、イヌリンもしくはマルトースの摂取に対象者の負担を要する。しかし、疾患に対する一般的な診療と比較して来院回数は変わらず、試験対象者の症状や治療経過に影響を与えない。

10.2.2. 起こりうる副作用等

試験食品または対照食品を摂取することによる有害事象として、イヌリン摂取による下痢、軟便、腹部膨満感が挙げられる。また、内容物による食物アレルギーが考え得るが、いずれもリスクは低い。また、1回あたりの採血量は介入前に40ml（ヒト末梢血単核細胞（PBMC）を単離し、フローサイトメトリーによる解析を行うため。）、介入直後に60ml（フローサイトメトリーによる解析に加え、PBMCより更にTregを単離し培養実験を行うため。）であり、貧血を来たすリスクは極めて低いと考える。

11. 有害事象が発生した際の対応

有害事象の定義を以下に示す。

有害事象:

実施された試験との因果関係の有無を問わず、試験対象者に生じた全ての好ましくない又は意図しない傷病もしくはその徴候（臨床検査値の異常を含む。）とする。

11.1. 有害事象発生時の試験対象者への対応

有害事象を認めたときは、試験担当者は直ちに適切な処置を行うとともに、診療録等に記載する。また、試験食品の投与を中止した場合や、有害事象に対する治療が必要となった場合には、試験対象者にその旨伝える。

11.2. 重篤な有害事象の報告

該当なし

12. 試験対象者に対する健康被害発生時の対応と補償等

本試験の実施に伴い、試験対象者に健康被害が生じた場合は、治療にかかる費用は健康保険を使用する。

13. 個々の試験対象者における中止基準と試験中止時等の対応

13.1. 個々の試験対象者における中止基準

試験責任者は、試験期間中に下記に該当する試験対象者が発生した場合には、当該試験対象者に対する試験を中止する。

1) 試験対象者より中止の申し入れがあった場合
2) 試験対象者の都合により試験が中断された場合（転居、転医・転院、多忙、追跡不能等）
3) 試験開始後、試験対象者が対象症例ではないことが判明した場合
4) 偶発的な事故が発生した場合
5) 有害事象が発現し（原疾患の増悪、合併症の増悪又は偶発症を含む）、試験責任者が中止すべきと判断した場合。
6) 効果不十分又は症状悪化のため、試験の継続が困難となった場合
7) 本試験実施計画書から重大な逸脱があり評価不能と判断される場合
8) 試験対象者が試験担当者の指示どおり摂取していないことが判明した場合
9) その他、試験責任者が試験の継続を困難と判断し中止が妥当と判断した場合

13.2. 試験の中止・中断、終了

13.2.1. 試験の中止・中断

試験担当者は、以下の事項に該当する場合は、試験実施継続の可否を検討する。

1) 安全性及び有効性に関する事項及びその他の重要な情報を知りえたとき。
2) 試験対象者の組み入れが困難で、予定症例数に達することが極めて困難であると判断されたとき。
3) 予定症例数又は予定期間に達する前に、試験の目的が達成されたとき。
4) 審査委員会により、実施計画等の変更の指示があり、これを受入れることが困難と判断されたとき。

試験責任者は、審査委員会により中止の勧告あるいは指示があった場合は、試験を中止する。また、試験の中止又は中断を決定した時は、速やかに倫理審査委員会及び試験機関の長にその理由とともに文書又は電磁的方法で報告する。

13.2.2. 試験の終了

試験責任者は、試験を終了した時は、その旨及び試験結果の概要を文書又は電磁的方法により遅滞なく倫理審査委員会及び試験機関の長に報告する。

14. 目標症例数とその設定根拠、及び統計解析方法

14.1. 目標症例数とその設定根拠

症例数の設定にあたっては、過去の健常者への介入研究において、機能性大麦やイヌリンの摂取により酪酸産生菌の割合が 2～5%ポイント程度増加することが報告されている。
本研究では、疾患群における腸内細菌叢の dysbiosis が補正されることによる反応性の高さを考慮し、イヌリン群で平均 12.5%の増加、プラセボ群で平均 2.5%の増加を仮定し、群間差 10%ポイントを期待値として設定した。この差と共通標準偏差 15%に基づき、Cohen の効果量を約 0.67 とし、検出力(Power)0.8、有意水準(α)0.05(両側検定)で G*Power(ver.3.1.9)を用いて症例数を算出した結果、各群 36 名が必要とされた。脱落者(約 10%)を見込み、イヌリン群 40 名、プラセボ群 40 名の計 80 名を目標症例数として設定する。

＜目標症例数＞
口腔扁平苔癬
各群 40 例、計 80 例
九州大学病院 60 例
岡山大学病院 20 例

14.2. 統計解析方法

主要評価項目として「酪酸産生菌割合の変化量」を設定し、イヌリン群とプラセボ群における群間差を比較することで、介入効果を検証する。解析にあたっては、各被験者について介入前後の酪酸産生菌割合の差(Δ 値)を算出し、群間の平均変化量を比較する。正規性の有無に応じて、独立 2 群の t 検定または Mann-Whitney の U 検定を用いる。また、年齢・性別などの背景因子が主要評価項目に影響を与える可能性があるため、必要に応じてこれらを共変量として加えた共分散分析(ANCOVA)を補助的に実施する。

補助評価項目として、腸内細菌叢の構成、唾液中免疫マーカー、および便中短鎖脂肪酸(SCFA)濃度(酪酸、酢酸、プロピオン酸)を評価する。腸内細菌叢の多様性指標には Shannon 指数および UniFrac 距離を用い、対応のある t 検定もしくは Wilcoxon 符号付順位検定、PERMANOVA により介入前後の変化を解析する。免疫マーカーについては、対応のある t 検定または Wilcoxon 符号付順位検定を用いて群内変化を評価する。いずれも、群間比較には正規性の有無に応じて、独立 2 群の t

検定または Mann-Whitney の U 検定を用いる。SCFA 濃度については、時間と群の交互作用を検討するため、反復測定分散分析（RM-ANOVA）または線形混合モデルを用いる。

有意水準は両側検定で 0.05 とし、p 値が 0.05 未満の場合を統計的に有意と判断する。統計解析には R（ver.4.3.1）を使用し、腸内細菌叢の構成解析には QIIME2 および LEfSe を併用する予定である。

15. 倫理的事項

15.1. 試験対象者に対する倫理的配慮

本試験のすべての担当者は、「ヘルシンキ宣言」（2024 年・第 75 回世界医師会ヘルシンキ総会改訂版）及び「人を対象とする生命科学・医学系試験に関する倫理指針」（令和 3 年文部科学省・厚生労働省・経済産業省告示第 1 号）を遵守して実施する。

15.2. インフォームド・コンセント等の取得

試験担当者は、倫理審査委員会で承認の得られた同意説明文書を試験対象者に渡し、文書及び口頭による十分な説明を行い、試験対象者の自由意思による同意を文書で取得する。

16. 個人情報等の取扱い

試験実施に係る試料・情報を取扱う際は、特定の個人を直ちに判別できる情報（氏名、住所、診療録番号等）は利用せず、試験対象者とは無関係の番号（試験対象者識別コード）を付して仮名加工情報として管理し、試験対象者の秘密保護に十分配慮する。試験対象者識別コードリストは、試験責任者が厳重に保管するよう監督する。試料・情報を試験事務局等の関連機関に送付する場合はこの番号を使用し、試験対象者の氏名、生年月日などの情報が院外に漏れないよう十分配慮する。また、試験責任者等が本試験で得られた情報を公表する際は、試験対象者を特定できる情報を含まないようにする。試験の目的以外に、試験で得られた試験対象者の試料・情報を使用しない。個人情報管理責任者は九州大学大学院歯学研究院口腔顎顔面外科学分野　教授　森山雅文とする。

共同研究機関で取得された情報は、九州大学へファイル共有システム Proself を用いてデータの受け渡しを行う。受領後は速やかに当科の専用 PC に移管し、元データは提供元施設にて適切に破棄または保管される。

17. 試料・情報の保管及び廃棄の方法

試験責任者は、定められた保管方法に従って試験担当者が試料および情報等（試験機関の長からの通知文書、各種申請書・報告書の控え、試験計画書、同意説明文書、試験対象者識別コードリスト、署名済み同意書、症例報告書等の控え、原資料、その他データの信頼性を保証するのに必要な書類又は記録等を含む）を適切に保管するよう指導し、試料および情報等の漏えい、混交、盗難、紛失等が起こらないよう必要な管理を行う。

採取した便、血液は、原則として当該試験の終了について報告された日から 5 年を経過した日又は当該試験の結果の最終の公表について報告された日から 5 年を経過した日のいずれか遅い日までの期間、顎顔面外科学分野研究室で冷凍保管する。

また、本試験で得られた情報等は、顎顔面外科学分野医局内の特定のキャビネットに施錠した状態で可能な限り長期間保管し、原則として当該試験の終了について報告された日から 10 年を経過した日又は当該試験の結果の最終の公表について報告された日から 10 年を経過した日のいずれか遅い日までの期間、適切に保管する。

試料を廃棄する際は、個人情報に注意し通常の医療廃棄物として、院内の廃棄手順に従って廃棄し、情報等を廃棄する際は、個人情報に注意して、紙媒体はシュレッダー処理・焼却、電子ファイルは専用のソフトウェア等により復元不可能な状態にするものとする。

保管責任者:森山　雅文

なお、共同研究機関においては、各機関の手順に従って適切に保管・廃棄する。

18. 試料・情報の二次利用について

本試験で得られた試験対象者の試料・情報は、同意を受ける時点では特定されない将来の試験のために用いる可能性がある。二次利用する試料、情報等は将来新たに計画・実施される試験が倫理審査委員会で承認された後に利用する。

その場合には、試験対象者に通知し、又は公開し、試験が実施されることについて、試験対象者が拒否できる機会を保障した上で実施する。

19. 試験機関の長への報告

1）試験の倫理的妥当性・科学的合理性を損なう事実若しくは情報又は損なうおそれのある情報であって試験の継続に影響を与えると考えられるものを得た場合には、臨床試験倫理審査委員会事務局に報告するとともに、遅滞なく試験機関の長に報告し、必要に応じて、試験を停止し、若しくは中止し、又は試験計画書を変更する。

2）試験の実施の適正性若しくは試験結果の信頼を損なう事実若しくは情報又は損なうおそれのある情報を得た場合には、臨床試験倫理審査委員会事務局に報告するとともに、速やかに試験機関の長に報告する。また、必要に応じて、試験を停止し、若しくは中止し、又は試験計画書を変更する。

3）試験開始後 1 年毎の進捗状況、及び、終了時（停止もしくは中止、又は変更の場合を含む）には、臨床試験倫理審査委員会事務局に報告するとともに、試験機関の長にその旨及び概要を報告する。

4）その他、試験実施に伴う重篤な有害事象が発生した場合には、当該状況について臨床試験倫理審査委員会事務局に報告するとともに、試験機関の長に報告する（上記「11. 有害事象が発生した際の対応」項に当該内容を記載)。

20. データの収集およびデータマネジメント

本試験は研究分担者（南部孝樹）がデータ収集及びデータマネジメントを適切に行う。

21. 試験対象者等及びその関係者からの相談等への対応

試験対象者等及びその関係者からの相談については、下記の相談窓口にて対応する。

【相談窓口】

試験代表者　九州大学歯学研究院口腔顎顔面外科学分野　教授　森山雅文

〒812-8582　福岡県福岡市東区馬出3-1-1

電話：092-642-6447（内線：3073）

22. 個々の試験対象者における試験結果の取扱い

本試験により得られた結果のうち、通常診療で実施する検査の結果については通常診療と同様に試験対象者に説明する。通常診療以外の結果については原則として試験対象者に個別に結果を伝えない。試験対象者からの希望があれば腸内細菌叢に占める酪酸産生菌割合のみ伝える。

23. 試験成果の発表方法

本試験成果の発表については、国際・国内学会での口頭発表、及び、論文での学術雑誌への発表もって公表とする。

試験成果を公表する際は、試験対象者等及びその関係者の人権又は試験担当者等及びその関係者の権利利益の保護のために必要な措置を講じた上で行う。

24. 試験者等の利益相反に関する状況

本試験に用いるイヌリンはオランダ SENSUS によって製造され、伊藤忠商事株式会社により輸入、バブルスター（株）に販売されたのち、（株）ウメケンにより個包装化され、バブルスター（株）より無償提供される。また、マルトースについては、ナガセヴィータ（株）により製造され、（株）ウメケンにより個包装化されたものを、バブルスター（株）より無償提供される。本試験は、口腔顎顔面外科学分野の研究費により実施し、外部の企業からの資金提供は受けない。バブルスター（株）、（株）ウメケンおよび伊藤忠商事株式会社は、食品に関する情報は提供するが、試験者が独立して計画し実施するものであり、試験の実施、解析、報告に係わることはない。なお、バブルスター（株）および伊藤忠商事株式会社との間では、試験食品提供に関する契約書を正式に交わしており、当該契約において研究の独立性および宣伝利用の禁止が明文化されている。

また、本試験の試験責任者および試験分担者は、利益相反マネジメント委員会に必要事項を申告し、その審査と承認を得るものとする。共同研究機関の試験者の利益相反に関しては、各機関の規定等に基づき厳密に管理する。

25. 特許権等の知的財産権について

本試験の成果に係る特許権等の知的財産権は九州大学に帰属する。

26. 試験の実施体制（統計解析、委託機関等も含む）

26.1 試験代表者

九州大学大学院歯学研究院口腔顎顔面外科学分野　教授　森山雅文

26.2. 試験責任者及び試験分担者等

試験責任者：

九州大学大学院歯学研究院口腔顎顔面外科学分野　教授　森山雅文

試験実施担当者：

九州大学大学院歯学研究院口腔顎顔面外科学分野　助教　金子直樹

九州大学大学院歯学研究院口腔顎顔面外科学分野　助教　陳鵠

九州大学病院顎口腔外科　医員　横溝志保

九州大学大学院歯学府顎顔面腫瘍制御学分野　大学院生　末吉智貴

九州大学大学院歯学府口腔顎顔面外科学分野　大学院生　城戸孟

九州大学大学院歯学府顎顔面腫瘍制御学分野　大学院生　鄢麗晶
九州大学大学院歯学府口腔顎顔面外科学分野　大学院生　田中瑞歩
九州大学大学院歯学府口腔顎顔面外科学分野　大学院生　藤野晴絵
九州大学大学院歯学府口腔顎顔面外科学分野　大学院生　久保慧乃葉
九州大学大学院歯学府口腔顎顔面外科学分野　大学院生　南部孝樹
九州大学大学院歯学府口腔顎顔面外科学分野　大学院生　静間龍成
九州大学大学院歯学府口腔顎顔面外科学分野　大学院生　濵田真悠子

試験事務局:
担当者:　九州大学大学院歯学研究院口腔顎顔面外科学分野　助教　金子直樹
九州大学大学院歯学府口腔顎顔面外科学分野　大学院生　南部孝樹
連絡先電話:　092-642-6452(PHS:2223, 3084)
メールアドレス:　kaneko@dent.kyushu-u.ac.jp
nambu.koki.054.s.kyushu-u.ac.jp

データマネジメント実施機関:
九州大学歯学研究院口腔学顔面外科学分野
〒812-8582　福岡県福岡市東区馬出3-1-1
電話：092-642-6447

統計解析責任者:
九州大学大学院歯学研究院口腔顎顔面外科学分野　助教　金子直樹
〒812-8582　福岡県福岡市東区馬出3-1-1
電話：092-642-6447（PHS：2223）

便中有機酸測定施設:
タカラバイオ株式会社
代表取締役社長　宮村毅
〒525-0058
滋賀県草津市野路東七丁目 4 番 38 号
電話:077-565-6920

共同研究機関:
岡山大学病院
〒700-8558　岡山市北区鹿田町 2-5-1
電話:086-235-6702
本研究における役割:研究対象者のリクルート、同意取得、情報・試料の取得・提供
【試験責任者】
岡山大学学術研究院医歯薬学域口腔学顔面外科学分野　教授　伊原木聰一郎
【試験分担者】
岡山大学学術研究院医歯薬学域口腔学顔面外科学分野　助教　岩田　英治

27. その他:試験に関する情報公開(ホームページ)

この試験の概要は、「人を対象とする生命科学・医学系研究に関する倫理指針」に従い、試験を開始する前に公開データベースである大学病院医療情報ネットワーク研究センター　臨床試験登録システム

（UMIN-CTR）に登録し、試験計画書の変更及び試験の進み具合に応じて登録内容を更新する。試験を終了したときは、試験の結果を登録する。

28. 参考資料・文献リスト

1. Yamauchi, M., et al. (2017). Myeloid dendritic cells stimulated by thymic stromal lymphopoietin promote Th2 immune responses and the pathogenesis of oral lichen planus. PLoS ONE, 12(9), e0184942. https://doi.org/10.1371/journal.pone.0184942
2. Miyahara, Y., et al. (2023). Toll-like receptor 9-positive plasmacytoid dendritic cells promote Th17 immune responses in oral lichen planus stimulated by epithelium-derived cathepsin K. Scientific Reports, 13(1), 19320. https://doi.org/10.1038/s41598-023-46090-3
3. Atarashi, K., et al. (2011). Induction of colonic regulatory T cells by indigenous Clostridium species. Science, 331(6015), 337-341. https://doi.org/10.1126/science.1198469
4. Ivanov, I. I., et al. (2009). Induction of intestinal Th17 cells by segmented filamentous bacteria. Cell, 139(3), 485-498. https://doi.org/10.1016/j.cell.2009.09.033
5. Lee, Y. K., et al. (2011). Proinflammatory T-cell responses to gut microbiota promote experimental autoimmune encephalomyelitis. Proceedings of the National Academy of Sciences of the United States of America, 108(Suppl 1), 4615-4622. https://doi.org/10.1073/pnas.1000082107
6. Wu, H. J., et al. (2010). Gut-residing segmented filamentous bacteria drive autoimmune arthritis via T helper 17 cells. Immunity, 32(6), 815-827. https://doi.org/10.1016/j.immuni.2010.06.001
7. Yokomizo, S., et al. (2024). Dysbiosis of the gut microbiome may contribute to the pathogenesis of oral lichen planus through Treg dysregulation. Journal of Oral Biosciences, 66(1), 1-10. https://doi.org/10.1016/j.job.2023.12.001
8. Liu, Q., et al. (2022). Altered faecal microbiome and metabolome in IgG4-related sclerosing cholangitis and primary sclerosing cholangitis. Gut, 71(5), 899-909. https://doi.org/10.1136/gutjnl-2021-326925
9. Shen, Y., et al. (2024). Association between primary Sjögren's syndrome and gut microbiota disruption: A systematic review and meta-analysis. Clinical Rheumatology, 43(2), 603-619. https://doi.org/10.1007/s10067-023-06754-x
10. Baba, Y., et al. (2025). Enhancement of carbohydrate metabolism by probiotic and prebiotic intake promotes short-chain fatty acid production in the gut microbiome: A randomized, double-blind, placebo-controlled crossover trial. Bioscience, Biotechnology, and Biochemistry. https://doi.org/10.1093/bbb/zbaf071

Study Protocol

Evaluation of Changes in the Gut Microbiota Induced by Inulin Intake in Patients with Oral Lichen Planus

(Placebo-controlled, double-blind, randomized, parallel-group comparative trial)

Principal investigator: Masafumi Moriyama

Institution: Section of Oral and Maxillofacial Surgery, Department of Oral and Maxillofacial Diseases, Faculty of Dental Science, Kyushu University

Version: 1.1

Date of preparation: July 7, 2026

Table of Contents

0. Summary

1. Study title

2. Objective

3. Background and significance

4. Selection of study participants

5. Study methods

6. Schedule and study period

7. Post-study care

8. Outcomes

9. Financial burden or honoraria for study participants

10. Anticipated benefits and disadvantages

11. Management of adverse events

12. Management of and compensation for health damage to study participants

13. Discontinuation criteria for individual participants and actions at discontinuation

14. Target sample size, rationale, and statistical analysis methods

15. Ethical matters

16. Handling of personal information

17. Storage and disposal of specimens and information

18. Secondary use of specimens and information

19. Reporting to the head of the study institution

20. Data collection and data management

21. Response to consultations from study participants and related persons

22. Handling of individual study results

23. Method of publication of study results

24. Conflicts of interest of investigators and related parties

25. Intellectual property rights, including patent rights

26. Study implementation system, including statistical analysis and contracted organizations

27. Other: public disclosure of study information

28. References

0. Summary

0.1 Study Title

Evaluation of Changes in the Gut Microbiota Induced by Inulin Intake in Patients with Oral Lichen Planus.

0.2 Objective

In recent years, dysbiosis of the gut microbiota has been reported to be associated with the development of autoimmune diseases, inflammatory bowel disease, and allergic diseases. In the field of oral medicine, we have shown that the gut microbiota of patients with oral lichen planus is characterized by dysbiosis involving a reduction in butyrate-producing bacteria. Inulin is a soluble dietary fiber and acts as a prebiotic that increases butyrate-producing bacteria in the gut. The objective of this study is to determine whether intake of inulin by patients with oral lichen planus increases the proportion of butyrate-producing bacteria in the gut microbiota.

0.3 Participants: Eligibility and Exclusion Criteria

Eligibility criteria:

- Patients diagnosed with oral lichen planus by a dentist.

- Men and women aged 18 years or older.

- Individuals who, after receiving a sufficient explanation regarding participation in this study, have understood the explanation adequately and provided written informed consent of their own free will.

Exclusion criteria:

- Individuals who have used antibiotics within the previous 3 months.

- Individuals who have been hospitalized within the previous 3 months.

- Individuals who refuse to participate in this study.

- Pregnant patients or patients who may be pregnant.

- Individuals with food allergy to inulin or to Asteraceae plants used as raw materials.

- Any other individual judged by the principal investigator to be inappropriate as a study participant.

Individuals who use antibiotics during the study will also be excluded from the study.

0.4 Study Design

This study is planned as a placebo-controlled, double-blind, randomized, parallel-group comparative trial.

0.5 Outcomes

Primary outcome:

Change in the proportion of butyrate-producing bacteria in the gut microbiota before and after inulin intake.

Secondary outcomes:

Changes before and after intake of functional inulin in the following items:

- Differences in the gut microbiota.

- Blood test data, including white blood cell count, eosinophil count, IgE level, and specific IgE level.

- Disease symptoms, including extent and severity of mucositis.

- Gut microbiota and fecal organic acid concentrations: detailed analytical results for gut bacteria and substances produced by gut bacteria.

- Peripheral blood regulatory T-cell count: analyzed using additional blood samples.

- Differences in salivary microbiota.

0.6 Target Number of Registered Participants

Oral lichen planus: 80 participants.

0.7 Study Period

From the date of approval by the Clinical Research Ethics Committee of Kyushu University Hospital until March 31, 2031.

0.8 Schema

The study schema is shown in the approved Japanese protocol.

1. Study Title

Evaluation of Changes in the Gut Microbiota Induced by Inulin Intake in Patients with Oral Lichen Planus (placebo-controlled, double-blind, randomized, parallel-group comparative trial).

2. Objective

The objective of this study is to determine whether intake of inulin by patients with oral lichen planus increases the proportion of butyrate-producing bacteria in the gut microbiota and to clarify the presence or absence of dysbiosis.

3. Background and Significance

Oral lichen planus is a disease that causes chronic inflammation of the oral mucosa due to abnormalities of the immune system. It is encountered relatively frequently in dental practice; however, much of its etiology and pathogenesis remains unclear, and no curative treatment has been established. In oral lichen planus, CD4-positive T-cell infiltration into the lesion is known, and we have also reported its involvement.

More than 1,000 species and more than 40 trillion bacteria inhabit the human intestinal tract and form the gut microbiota, which has a major effect on the health of the human host. In recent years, numerous reports have shown that abnormalities of the gut microbiota, or dysbiosis, are deeply involved in the pathophysiology of allergic diseases, autoimmune diseases, and inflammatory bowel disease.

In our previous studies, we confirmed that patients with oral lichen planus have lower gut microbial diversity than healthy controls and, in particular, a reduction in butyrate-producing bacteria. These bacteria induce regulatory T cells (Tregs) in the intestinal tract, and Tregs suppress excessive immune responses. Therefore, reduction of butyrate-producing bacteria may be associated with persistence of inflammation and chronicity of lesions in oral lichen planus. Previous studies have also shown a positive correlation between reduction of gut bacteria and attenuation of Tregs with immunosuppressive function.

Inulin is a soluble dietary fiber that acts as a prebiotic in the intestinal tract and improves the gut microbiota. In a randomized, double-blind, crossover trial of bifidobacteria and inulin intake in 120 healthy adults in Japan, 2 weeks of intake changed the gut microbiota and increased useful short-chain organic acids in stool, including butyric acid, acetic acid, and propionic acid. Therefore, this study aims to determine whether intake of inulin by patients with oral lichen planus, who are considered to have dysbiosis characterized by reduced butyrate-producing bacteria, can increase the proportion of butyrate-producing bacteria in the gut microbiota.

If this study confirms that inulin can increase butyrate-producing bacteria in patients with oral lichen planus, the findings may lead to the development of treatment for this disease, for which no curative therapy has been established. The social need for such treatment is considered high.

4. Selection of Study Participants

4.1 Eligibility Criteria

Patients who meet all of the following criteria will be included:

- Patients diagnosed with oral lichen planus by a dentist.

- Men and women aged 18 years or older.

- Individuals who, after receiving a sufficient explanation regarding participation in this study, have understood the explanation adequately and provided written informed consent of their own free will.

4.2 Exclusion Criteria

Patients meeting any of the following criteria will be excluded:

- Use of antibiotics within the previous 3 months.

- Hospitalization within the previous 3 months.

- Refusal to participate in this study.

- Pregnancy or possible pregnancy.

- Food allergy to inulin or to Asteraceae plants used as raw materials.

- Any other condition judged by the principal investigator to make the individual inappropriate as a study participant.

Individuals who use antibiotics during the study will also be excluded from the study.

5. Study Methods

5.1 Overview

5.1.1 Study Design

Placebo-controlled, double-blind, randomized, parallel-group comparative trial.

5.1.2 Study Outline

For patients from whom consent has been obtained, eligibility and exclusion criteria will be reconfirmed. Participants will then be randomly assigned to the inulin intake group or placebo intake group based on a prepared allocation table. At the start of the intervention, blood collection (40 mL), stool collection, and saliva collection (5 mL) will be performed. In both groups, participants will consume one sachet (4 g) of the study food twice daily for a total of 4 weeks. During the intake period, participants will complete a daily dietary questionnaire. At the end of the intervention, blood collection (60 mL), stool collection, and saliva collection (5 mL) will be performed. Stool collection and saliva collection (5 mL) will also be performed 1 month after the end of the intervention.

5.1.3 Planned Participation Period for Each Participant

The intake period is 4 weeks, with a 2-week pre-observation period and a 4-week post-observation period.

5.2 Registration Method

The principal investigator or subinvestigators (hereafter, study personnel) will assign each participant a participant identification code, confirm eligibility, and register the case. Required information will be entered in the participant identification code list retained by the principal investigator.

5.3 Allocation Method

The allocation manager will prepare in advance an allocation table for 80 cases for random assignment of the study food and will store it appropriately. After consent is obtained from a participant, the principal investigator or a subinvestigator will promptly inquire with the allocation manager regarding assignment of the study treatment for that participant. Based on the allocation table prepared in advance, the allocation manager will notify study personnel of the study treatment listed in the allocation table in ascending order. When notification of allocation is made, the allocation manager will promptly enter and store the registration date, date of consent, identification code of the registered participant, and name of the study personnel who registered the participant in the allocation record.

The person responsible for blinding the study foods will confirm that the study foods cannot be distinguished. This person will prepare a correspondence table consisting of serial numbers, placebo and study foods (inulin and maltose) in random order, and study food allocation codes (symbols and numbers). Based on this correspondence table, the study foods will be labeled with

study food allocation code names. The correspondence table will be stored by the person responsible for study food blinding until unblinding and will not be disclosed to others. Except when unblinding is performed for an individual case as an emergency measure, unblinding will not occur until the study has ended and the inclusion or exclusion of each case has been finalized.

5.4 Administration of Study Food

5.4.1 Overview of Study Food

Study product:

Product name: Inulia® IQ.

Raw material: Inulin.

Content: 4 g per sachet.

Storage: Room temperature.

Manufacturer: Sensus, Netherlands.

Importer: Itochu Corporation, 2-5-1 Kita-Aoyama, Minato-ku, Tokyo.

Individual packaging: Umeken Co., Ltd., 3-16 Kurosaki-cho, Kita-ku, Osaka-shi, Osaka.

Provider: Babblestar Co., Ltd., 1-15-15 Daikan, Yamato-shi, Kanagawa.

Control food:

Placebo: Sanmalt Shiro.

Raw material: Maltose.

Content: 4 g per sachet.

Storage: Room temperature.

Manufacturer: Nagase Viita Co., Ltd., 1-1-3 Shimoishii, Kita-ku, Okayama-shi, Okayama.

Individual packaging: Umeken Co., Ltd., 3-16 Kurosaki-cho, Kita-ku, Osaka-shi, Osaka.

Provider: Babblestar Co., Ltd., 1-15-15 Daikan, Yamato-shi, Kanagawa.

5.4.2 Administration Method

After the pre-observation period, participants will consume either inulin or placebo according to allocation. The administration period will be 4 weeks from the start of intake. Participants will consume 4 g twice daily, for a total of 2 sachets (8 g) per day. The dose was selected based on previous research showing improvement of the intestinal environment with intake of 8 g of inulin.

5.4.3 Rules Regarding Concomitant Drugs and Therapies

The amount of inulin contained in common foods such as onions and garlic is small, and the effect of ordinary intake is extremely limited; therefore, these foods will not be restricted. In contrast, intake of inulin-containing supplements and fortified foods will be prohibited during the study period because such products may substantially change the amount of inulin consumed. Treatment for oral lichen planus during the study period, including topical steroid application, may be continued.

5.4.4 Rules Regarding Dose Reduction and Drug Holidays

Not applicable.

6. Schedule and Study Period

The observations, examinations, and evaluations specified in the study schedule will be conducted according to the schedule shown in the approved Japanese protocol.

Registration period: From the approval date by the Clinical Research Ethics Committee of Kyushu University Hospital to March 31, 2029.

Follow-up period: 10 weeks from the date of case registration.

Study period: From the approval date to March 31, 2031.

7. Post-study Care

Not applicable.

8. Outcomes

Primary outcome:

Change in the proportion of butyrate-producing bacteria in the gut microbiota before and after inulin intake.

Secondary outcomes:

Changes before and after intake of functional inulin in the following items:

- Differences in the gut microbiota.

- Blood test data, including white blood cell count, eosinophil count, IgE level, and specific IgE level.

- Disease symptoms, including extent and severity of mucositis.

- Gut microbiota and fecal organic acid concentrations.

- Peripheral blood regulatory T-cell count, analyzed using additional blood samples.

- Differences in salivary microbiota.

The gut microbiota and fecal organic acid concentration outcomes refer to detailed analytical results for gut bacteria and substances produced by gut bacteria. In this study, collected fecal samples will be anonymized and analysis will be outsourced to Takara Bio Inc.

9. Financial Burden or Honoraria for Study Participants

In this study, analysis of the gut microbiota, measurement of fecal organic acid concentrations, and measurement of Treg counts will be covered by research funds of the department to which the principal investigator belongs. Other procedures will be performed within ordinary insured medical care; therefore, participation in the study will not generate additional costs for participants. As an honorarium, a book gift card worth 10,000 yen will be provided for each visit (at the start of the intervention, at the end of the intervention, and 1 month after the end of the intervention; three visits in total). If a participant discontinues the study before completion, the honorarium will be provided for the visits completed up to that point; no honorarium will be provided for visits not completed.

10. Anticipated Benefits and Disadvantages

10.1 Anticipated Benefits

Inulin or maltose will be provided free of charge. Participants assigned to the control group by randomization may receive 4 weeks of inulin after the study period if they wish. The study results may also contribute to future advances in medicine.

10.2 Anticipated Disadvantages

10.2.1 Burden of Invasiveness and Related Procedures

This study involves collection of specimens during routine clinical care. Therefore, participants will bear the burden of blood collection, stool collection, and intake of inulin or maltose for study purposes. However, compared with ordinary clinical care for the disease, the number of hospital visits will not change, and the study will not affect participants' symptoms or treatment course.

10.2.2 Possible Adverse Reactions

Adverse events associated with intake of the study food or control food may include diarrhea, soft stools, and abdominal bloating due to inulin intake. Food allergy to the contents is also possible, although the risk is considered low. The blood volume collected at one time will be 40 mL before the intervention, for isolation of human peripheral blood mononuclear cells and analysis by flow cytometry, and 60 mL immediately after the intervention, for flow cytometric analysis and additional isolation of Tregs from peripheral blood mononuclear cells for culture experiments. The risk of anemia is considered extremely low.

11. Management of Adverse Events

Adverse event is defined as any unfavorable or unintended disease, sign, or symptom, including abnormal laboratory values, occurring in a study participant, regardless of whether there is a causal relationship with the study conducted.

11.1 Response to Participants When Adverse Events Occur

When an adverse event is observed, study personnel will immediately provide appropriate treatment and record the event in the medical record. If administration of the study food is discontinued or treatment for the adverse event becomes necessary, the participant will be informed accordingly.

11.2 Reporting of Serious Adverse Events

Not applicable.

12. Management of and Compensation for Health Damage to Study Participants

If health damage occurs in a study participant in association with implementation of this study, the costs of treatment will be covered using health insurance.

13. Discontinuation Criteria for Individual Participants and Actions at Discontinuation

13.1 Discontinuation Criteria for Individual Participants

The principal investigator will discontinue the study for a participant if any of the following occur during the study period:

1. The participant requests discontinuation.

2. The study is interrupted for reasons attributable to the participant, such as moving, transfer to another physician or hospital, being too busy, or being lost to follow-up.

3. After starting the study, it is found that the participant is not an eligible case.

4. An accidental event occurs.

5. An adverse event occurs, including worsening of the primary disease, worsening of a complication, or an incidental disease, and the principal investigator judges that discontinuation is necessary.

6. Continuation of the study becomes difficult due to insufficient effect or worsening of symptoms.

7. There is a major deviation from this study protocol and evaluation is judged impossible.

8. It is found that the participant has not consumed the study food as instructed by study personnel.

9. The principal investigator otherwise judges that continuation of the study is difficult and that discontinuation is appropriate.

13.2 Study Suspension, Interruption, and Completion

13.2.1 Study Suspension or Interruption

Study personnel will consider whether study continuation is possible if any of the following occur:

- Important information regarding safety, efficacy, or other matters becomes available.

- Enrollment of participants is difficult, and it is judged extremely difficult to reach the planned sample size.

- The study objective is achieved before the planned sample size or planned period is reached.

- The review committee instructs changes to the implementation plan or related documents, and it is judged difficult to accept the instruction.

If the review committee recommends or instructs suspension, the principal investigator will suspend the study. If suspension or interruption of the study is decided, the reason will be promptly reported to the ethics review committee and the head of the study institution in writing or by electromagnetic means.

13.2.2 Study Completion

When the study is completed, the principal investigator will report that fact and a summary of the study results to the ethics review committee and the head of the study institution without delay in writing or by electromagnetic means.

14. Target Sample Size, Rationale, and Statistical Analysis Methods

14.1 Target Sample Size and Rationale

For sample size setting, previous intervention studies in healthy individuals have reported that intake of functional barley or inulin increases the proportion of butyrate-producing bacteria by approximately 2 to 5 percentage points. In this study, considering the potentially greater responsiveness associated with correction of dysbiosis in the disease group, we assumed a mean increase of 12.5% in the inulin group and 2.5% in the placebo group, and set the expected between-group difference at 10 percentage points. Based on this expected difference and a common standard deviation of 15%, Cohen's effect size was set at approximately 0.67. With power of 0.8 and a two-sided significance level of 0.05, sample size calculation using G*Power version 3.1.9 indicated that 36 participants per group would be required. Allowing for dropouts of approximately 10%, the target sample size was set at 40 participants in the inulin group and 40 participants in the placebo group, for a total of 80 participants.

Target sample size for oral lichen planus: 40 participants per group, 80 participants in total. Kyushu University Hospital will enroll 60 participants, and Okayama University Hospital will enroll 20 participants.

14.2 Statistical Analysis Methods

The primary outcome will be change in the proportion of butyrate-producing bacteria. The intervention effect will be assessed by comparing the between-group difference between the inulin group and the placebo group. For analysis, the difference in the proportion of butyrate-producing bacteria before and after the intervention will be calculated for each participant, and mean change will be compared between groups. Depending on normality, an independent two-sample t test or Mann-Whitney U test will be used. Because background factors such as age and sex may affect the primary outcome, analysis of covariance (ANCOVA) including these variables as covariates will be conducted as a supplementary analysis if necessary.

Supplementary outcomes will include gut microbiota composition, salivary immune markers, and fecal short-chain fatty acid concentrations, including butyrate, acetate, and propionate. For gut microbiota diversity indices, the Shannon index and UniFrac distance will be used, and changes before and after the intervention will be analyzed using paired t tests, Wilcoxon signed-rank tests, or PERMANOVA. Immune markers will be evaluated for within-group change using paired t tests or Wilcoxon signed-rank tests. For between-group comparisons, independent two-sample t tests or Mann-Whitney U tests will be used depending on normality. For short-chain fatty acid concentrations, repeated-measures analysis of variance or linear mixed models will be used to examine the interaction between time and group.

The significance level will be two-sided 0.05, and p values less than 0.05 will be considered statistically significant. Statistical analyses will be performed using R version 4.3.1. QIIME 2 and LEfSe are planned for use in the analysis of gut microbiota composition.

15. Ethical Matters

15.1 Ethical Considerations for Study Participants

All personnel involved in this study will conduct the study in compliance with the Declaration of Helsinki, as revised by the 75th World Medical Association General Assembly in 2024, and with the Ethical Guidelines for Medical and Biological Research Involving Human Subjects, Notification No. 1 of the Ministry of Education, Culture, Sports, Science and Technology; Ministry of Health, Labour and Welfare; and Ministry of Economy, Trade and Industry of Japan, 2021.

15.2 Obtaining Informed Consent

Study personnel will provide participants with the informed consent document approved by the ethics review committee, give sufficient written and oral explanation, and obtain written consent based on the participant's free will.

16. Handling of Personal Information

When handling specimens and information related to study implementation, information that can immediately identify a specific individual, such as name, address, and medical record number, will not be used. A number unrelated to the participant, the participant identification code, will be assigned and the data will be managed as pseudonymized information, with sufficient consideration for protection of participant confidentiality. The participant identification code list will be strictly stored under the supervision of the principal investigator. When specimens or information are sent to the study office or related organizations, this number will be used, and sufficient care will be taken to prevent information such as participant name and date of birth from being disclosed outside the hospital. When the principal investigator and others publish information obtained in this study, information that can identify participants will not be included. Specimens and information obtained in the study will not be used for purposes other than the study. The person responsible for personal information management is Professor Masafumi Moriyama, Section of Oral and Maxillofacial Surgery, Faculty of Dental Science, Kyushu University.

Information obtained at collaborating research institutions will be transferred to Kyushu University using the Proself file-sharing system. After receipt, the data will be promptly transferred to a dedicated computer in the department, and the original data will be appropriately destroyed or stored at the providing institution.

17. Storage and Disposal of Specimens and Information

The principal investigator will instruct study personnel to appropriately store specimens and information according to the prescribed storage method. These include notifications from the head of the study institution, copies of applications and reports, the study protocol, informed consent documents, participant identification code lists, signed consent forms, copies of case report forms, source documents, and other documents or records necessary to assure data reliability. Necessary management will be performed to prevent leakage, mixing, theft, loss, or similar incidents involving specimens and information.

Collected stool and blood will generally be frozen and stored in the laboratory of the Section of Oral and Maxillofacial Surgery until the later of 5 years after the date on which completion of the

study is reported or 5 years after the date on which final publication of the study results is reported.

Information and other records obtained in this study will be stored for as long as possible in a locked specific cabinet in the Section of Oral and Maxillofacial Surgery office. As a general rule, they will be appropriately stored until the later of 10 years after the date on which completion of the study is reported or 10 years after the date on which final publication of the study results is reported.

When specimens are disposed of, personal information will be protected and specimens will be discarded as ordinary medical waste according to hospital disposal procedures. When information is disposed of, personal information will be protected, paper documents will be shredded or incinerated, and electronic files will be rendered unrecoverable using dedicated software or similar methods. The person responsible for storage is Masafumi Moriyama. At collaborating research institutions, storage and disposal will be performed appropriately according to each institution's procedures.

18. Secondary Use of Specimens and Information

Specimens and information obtained from participants in this study may be used for future studies that are not specified at the time consent is obtained. Specimens and information for secondary use will be used only after newly planned and implemented future studies have been approved by an ethics review committee. In such cases, participants will be notified or information will be disclosed publicly, and participants will be given an opportunity to refuse implementation of the study.

19. Reporting to the Head of the Study Institution

1. If facts or information are obtained that compromise, or may compromise, the ethical validity or scientific rationality of the study and are considered to affect continuation of the study, the matter will be reported to the Clinical Research Ethics Committee office and to the head of the study institution without delay. If necessary, the study will be stopped or discontinued, or the study protocol will be amended.

2. If facts or information are obtained that compromise, or may compromise, the appropriateness of study implementation or the reliability of study results, the matter will be reported to the Clinical Research Ethics Committee office and promptly to the head of the study institution. If necessary, the study will be stopped or discontinued, or the study protocol will be amended.

3. Progress after study initiation will be reported annually to the Clinical Research Ethics Committee office, and at study completion, including suspension, discontinuation, or amendment, the fact and summary will be reported to the head of the study institution.

4. If any serious adverse event occurs in association with study implementation, the situation will be reported to the Clinical Research Ethics Committee office and to the head of the study institution, as described in Section 11, Management of Adverse Events.

20. Data Collection and Data Management

In this study, subinvestigator Koki Nambu will appropriately conduct data collection and data management.

21. Response to Consultations from Study Participants and Related Persons

Consultations from study participants and related persons will be handled through the following consultation desk.

Consultation desk:

Study representative: Professor Masafumi Moriyama, Section of Oral and Maxillofacial Surgery, Faculty of Dental Science, Kyushu University.

Address: 3-1-1 Maidashi, Higashi-ku, Fukuoka 812-8582, Japan.

Telephone: 092-642-6447, extension 3073.

22. Handling of Individual Study Results

Among the results obtained in this study, results of tests conducted as part of ordinary clinical care will be explained to participants in the same manner as ordinary clinical care. Results other than those of ordinary clinical care will generally not be individually communicated to participants. If a participant requests it, only the proportion of butyrate-producing bacteria in the gut microbiota will be communicated.

23. Method of Publication of Study Results

Study results will be made public through oral presentations at international and domestic academic conferences and through publication in academic journals. When study results are published, necessary measures will be taken to protect the human rights of participants and related persons and the rights and interests of study personnel and related persons.

24. Conflicts of Interest of Investigators and Related Parties

The inulin used in this study is manufactured by Sensus in the Netherlands, imported by Itochu Corporation, sold to Babblestar Co., Ltd., individually packaged by Umeken Co., Ltd., and provided free of charge by Babblestar Co., Ltd. Maltose is manufactured by Nagase Viita Co., Ltd., individually packaged by Umeken Co., Ltd., and provided free of charge by Babblestar Co., Ltd. This study will be conducted using research funds from the Section of Oral and Maxillofacial Surgery, and no financial support will be received from external companies. Babblestar Co., Ltd., Umeken Co., Ltd., and Itochu Corporation will provide information about the foods, but the investigators will independently plan and conduct the study, and these companies will not be involved in study implementation, analysis, or reporting. Formal contracts regarding provision of the study foods have been concluded with Babblestar Co., Ltd. and Itochu Corporation, and these contracts explicitly state the independence of the research and prohibit promotional use.

The principal investigator and subinvestigators of this study will declare necessary matters to the Conflict of Interest Management Committee and obtain its review and approval. Conflicts of interest for investigators at collaborating research institutions will be strictly managed according to the regulations of each institution.

25. Intellectual Property Rights, Including Patent Rights

Intellectual property rights, including patent rights related to the results of this study, will belong to Kyushu University.

26. Study Implementation System, Including Statistical Analysis and Contracted Organizations

26.1 Study Representative

Professor Masafumi Moriyama, Section of Oral and Maxillofacial Surgery, Faculty of Dental Science, Kyushu University.

26.2 Principal Investigator, Subinvestigators, and Related Personnel

Principal investigator:

Professor Masafumi Moriyama, Section of Oral and Maxillofacial Surgery, Faculty of Dental Science, Kyushu University.

Study personnel:

Naoki Kaneko, Assistant Professor, Section of Oral and Maxillofacial Surgery, Faculty of Dental Science, Kyushu University.

Hu Chen, Assistant Professor, Section of Oral and Maxillofacial Surgery, Faculty of Dental Science, Kyushu University.

Shiho Yokomizo, Medical Staff, Department of Oral and Maxillofacial Surgery, Kyushu University Hospital.

Tomoki Sueyoshi, Graduate Student, Section of Oral and Maxillofacial Oncology, Graduate School of Dental Science, Kyushu University.

Takeshi Kido, Graduate Student, Section of Oral and Maxillofacial Surgery, Graduate School of Dental Science, Kyushu University.

Lijing Yan, Graduate Student, Section of Oral and Maxillofacial Oncology, Graduate School of Dental Science, Kyushu University.

Mizuho Tanaka, Graduate Student, Section of Oral and Maxillofacial Surgery, Graduate School of Dental Science, Kyushu University.

Harue Fujino, Graduate Student, Section of Oral and Maxillofacial Surgery, Graduate School of Dental Science, Kyushu University.

Konoha Kubo, Graduate Student, Section of Oral and Maxillofacial Surgery, Graduate School of Dental Science, Kyushu University.

Koki Nambu, Graduate Student, Section of Oral and Maxillofacial Surgery, Graduate School of Dental Science, Kyushu University.

Ryusei Shizuma, Graduate Student, Section of Oral and Maxillofacial Surgery, Graduate School of Dental Science, Kyushu University.

Mayuko Hamada, Graduate Student, Section of Oral and Maxillofacial Surgery, Graduate School of Dental Science, Kyushu University.

Study office:

Naoki Kaneko, Assistant Professor, Section of Oral and Maxillofacial Surgery, Faculty of Dental Science, Kyushu University.

Koki Nambu, Graduate Student, Section of Oral and Maxillofacial Surgery, Graduate School of Dental Science, Kyushu University.

Telephone: 092-642-6452 (PHS: 2223, 3084).

Email: kaneko@dent.kyushu-u.ac.jp; nambu.koki.054.s.kyushu-u.ac.jp.

Data management institution:

Section of Oral and Maxillofacial Surgery, Faculty of Dental Science, Kyushu University.

3-1-1 Maidashi, Higashi-ku, Fukuoka 812-8582, Japan.

Telephone: 092-642-6447.

Responsible statistician:

Naoki Kaneko, Assistant Professor, Section of Oral and Maxillofacial Surgery, Faculty of Dental Science, Kyushu University.

3-1-1 Maidashi, Higashi-ku, Fukuoka 812-8582, Japan.

Telephone: 092-642-6447 (PHS: 2223).

Facility for fecal organic acid measurement:

Takara Bio Inc.

President and Representative Director: Tsuyoshi Miyamura.

7-4-38 Nojihigashi, Kusatsu, Shiga 525-0058, Japan.

Telephone: 077-565-6920.

Collaborating research institution:

Okayama University Hospital.

2-5-1 Shikata-cho, Kita-ku, Okayama 700-8558, Japan.

Telephone: 086-235-6702.

Role in this study: recruitment of study participants, obtaining consent, acquisition and provision of information and specimens.

Principal investigator at collaborating institution:

Professor Soichiro Ibaragi, Department of Oral and Maxillofacial Surgery, Graduate School of Medicine, Dentistry and Pharmaceutical Sciences, Okayama University.

Subinvestigator at collaborating institution:

Eiji Iwata, Assistant Professor, Department of Oral and Maxillofacial Surgery, Graduate School of Medicine, Dentistry and Pharmaceutical Sciences, Okayama University.

27. Other: Public Disclosure of Study Information

In accordance with the Ethical Guidelines for Medical and Biological Research Involving Human Subjects, the outline of this study will be registered in the University Hospital Medical Information Network Clinical Trials Registry (UMIN-CTR), a public database, before the start of the study. Registered information will be updated according to changes in the study protocol and study progress. When the study is completed, the study results will be registered.

28. References

1. Yamauchi M, et al. Myeloid dendritic cells stimulated by thymic stromal lymphopoietin promote Th2 immune responses and the pathogenesis of oral lichen planus. PLoS ONE. 2017;12(9):e0184942. https://doi.org/10.1371/journal.pone.0184942

2. Miyahara Y, et al. Toll-like receptor 9-positive plasmacytoid dendritic cells promote Th17 immune responses in oral lichen planus stimulated by epithelium-derived cathepsin K. Scientific Reports. 2023;13(1):19320. https://doi.org/10.1038/s41598-023-46090-3

3. Atarashi K, et al. Induction of colonic regulatory T cells by indigenous Clostridium species. Science. 2011;331(6015):337-341. https://doi.org/10.1126/science.1198469

4. Ivanov II, et al. Induction of intestinal Th17 cells by segmented filamentous bacteria. Cell. 2009;139(3):485-498. https://doi.org/10.1016/j.cell.2009.09.033

5. Lee YK, et al. Proinflammatory T-cell responses to gut microbiota promote experimental autoimmune encephalomyelitis. Proceedings of the National Academy of Sciences of the United States of America. 2011;108(Suppl 1):4615-4622. https://doi.org/10.1073/pnas.1000082107

6. Wu HJ, et al. Gut-residing segmented filamentous bacteria drive autoimmune arthritis via T helper 17 cells. Immunity. 2010;32(6):815-827. https://doi.org/10.1016/j.immuni.2010.06.001

7. Yokomizo S, et al. Dysbiosis of the gut microbiome may contribute to the pathogenesis of oral lichen planus through Treg dysregulation. Journal of Oral Biosciences. 2024;66(1):1-10. https://doi.org/10.1016/j.job.2023.12.001

8. Liu Q, et al. Altered faecal microbiome and metabolome in IgG4-related sclerosing cholangitis and primary sclerosing cholangitis. Gut. 2022;71(5):899-909. https://doi.org/10.1136/gutjnl-2021-326925

9. Shen Y, et al. Association between primary Sjogren's syndrome and gut microbiota disruption: a systematic review and meta-analysis. Clinical Rheumatology. 2024;43(2):603-619. https://doi.org/10.1007/s10067-023-06754-x

10. Baba Y, et al. Enhancement of carbohydrate metabolism by probiotic and prebiotic intake promotes short-chain fatty acid production in the gut microbiome: a randomized, double-blind, placebo-controlled crossover trial. Bioscience, Biotechnology, and Biochemistry. 2025. https://doi.org/10.1093/bbb/zbaf071
