# Supplementary material for: Study protocol: Effect of inulin supplementation on gut microbiota in patients with oral lichen planus — A double-blind, randomised, placebo-controlled parallel-group trial
Source: PLoS One. 2026 Aug 13;21(8):e0356180. doi: 10.1371/journal.pone.0356180 (PMC13472398; doi:10.1371/journal.pone.0356180)
Supplement: Supplementary file 3 [file pone.0356180.s003.docx]

Daily Dietary Record (Intervention Period)

Study ID : Response period:（	~	）

Please circle the foods you consumed.

| **Food category** | **Examples** | **/**  **(M)** | **/**  **(E)** | **/　(M)** | **/　(E)** | **/　(M)** | **/　(E)** | **/　(M)** | **/　(E)** | **/　(M)** | **/　(E)** | **/　(M)** | **/　(E)** | **/　(M)** | **/　(E)** |
| --- | --- | --- | --- | --- | --- | --- | --- | --- | --- | --- | --- | --- | --- | --- | --- |
|  | **Inulin** |  |  |  |  |  |  |  |  |  |  |  |  |  |  |
| **Staple foods** | **Rice, bread, noodles** |  |  |  |  |  |  |  |  |  |  |  |  |  |  |
| **Vegetables** | **Raw vegetables** |  |  |  |  |  |  |  |  |  |  |  |  |  |  |
|  | **Beans, potatoes, root vegetables** |  |  |  |  |  |  |  |  |  |  |  |  |  |  |
|  | **Mushrooms, seaweed** |  |  |  |  |  |  |  |  |  |  |  |  |  |  |
| **Soy products** | **Tofu, deep‑fried tofu, soy milk** |  |  |  |  |  |  |  |  |  |  |  |  |  |  |
| **Fruits** | **Fruits** |  |  |  |  |  |  |  |  |  |  |  |  |  |  |
| **Meat / Fish** | **Pork, chicken, beef, fish** |  |  |  |  |  |  |  |  |  |  |  |  |  |  |
| **Dairy products** | **Milk, cheese** |  |  |  |  |  |  |  |  |  |  |  |  |  |  |
| **Fermented foods** | **Yogurt, natto, kimchi, pickles** |  |  |  |  |  |  |  |  |  |  |  |  |  |  |
| **Processed foods** | **Snacks, soft drinks** |  |  |  |  |  |  |  |  |  |  |  |  |  |  |
