# Supplementary material for: Study protocol: Effect of inulin supplementation on gut microbiota in patients with oral lichen planus — A double-blind, randomised, placebo-controlled parallel-group trial
Source: PLoS One. 2026 Aug 13;21(8):e0356180. doi: 10.1371/journal.pone.0356180 (PMC13472398; doi:10.1371/journal.pone.0356180)
Supplement: Supplementary file 4 [file pone.0356180.s005.docx]

**Response to Reviewers**

**Manuscript ID:** PONE-D-26-25644

**Title:** Study protocol: Effect of inulin supplementation on gut microbiota in patients with oral lichen planus — a double-blind, randomised, placebo-controlled parallel-group trial

We would like to thank the Academic Editor, journal staff, and Reviewer #1 for their careful evaluation of our manuscript and constructive comments. We have revised the manuscript accordingly. The principal revisions are summarized below, followed by a point-by-point response.

# Summary of revisions

1. The planned sample size was revised from 60 to 80 participants in response to the reviewer's comment.
2. The sample size calculation was revised to clarify that approximately 36 participants per group are required before allowing for dropout and that 40 participants per group are planned after allowing for approximately 10% dropout.
3. The Data Availability Statement was revised to clarify that this protocol article reports no datasets, that raw 16S rRNA gene sequencing data from the completed trial will be deposited in the DDBJ Sequence Read Archive, and that the de-identified participant-level data and related materials underlying the completed trial findings will be made publicly available with the primary results publication.
4. The ethics statement was confirmed to appear only in the Methods section.
5. The ethics amendment increasing the target sample size to 80 participants was approved by the Clinical Research Ethics Committee of Kyushu University Hospital on 16 July 2026.
6. The recruitment feasibility statement and related descriptions were revised to align with the updated target sample size.
7. The reference list was revised to follow the PLOS author-list format. Published correction notices for the Furusawa et al. and Bolyen et al. articles were added, and the corresponding in-text citations were renumbered accordingly.
8. The Supporting Information captions were moved to the end of the manuscript after the References.

A point-by-point response follows.

# Journal Requirements

# Requirement 1. PLOS ONE style requirements

# Please ensure that your manuscript meets PLOS ONE's style requirements, including those for file naming.

**Response:** We have reviewed the PLOS ONE formatting requirements and revised the manuscript accordingly. Continuous line numbering and page numbering are present, Supporting Information captions are placed at the end of the manuscript, and separate clean and marked-up versions have been prepared for submission. We also updated the reported page numbers in the completed SPIRIT 2025 checklist (S1 Checklist) to correspond to the revised clean manuscript.

## Requirements 2–3. Data Availability Statement

The journal requested replacement of the previous author contact point, clarification of future data availability, and either public availability of the data underlying the findings or a justified request for an ethical or legal exemption.

**Response:** We have revised the Data Availability Statement. The revised statement clarifies that this protocol article does not report datasets generated or analysed during the current protocol stage. After completion of the trial and publication of the primary trial results, raw 16S rRNA gene sequencing data will be deposited in the DDBJ Sequence Read Archive. De-identified participant-level data, processed microbiome data, metadata, and a data dictionary underlying the completed trial findings will be made available as Supporting Information files. Accession numbers, DOIs, or other persistent identifiers will be provided in the primary results publication. Because the revised statement specifies public routes for the future datasets, the previous author contact point has been removed.

## Requirement 4. Ethics statement location

The journal requested that the ethics statement appear only in the Methods section.

**Response:** We confirmed that the ethics statement appears only in the Materials and methods section under 'Ethical considerations' and that no duplicate ethics statement appears elsewhere in the manuscript.

## Requirement 5. Competing Interests Statement

The journal requested an amended Competing Interests Statement in the cover letter that explicitly states the commercial source of in-kind support; addresses employment, consultancy, patents, products in development, marketed products, and other relevant relationships; and confirms adherence to PLOS ONE policies on sharing data and materials.

**Response:** We have included the amended Competing Interests Statement in the revised cover letter. The statement explicitly identifies the commercial in-kind support and confirms our adherence to PLOS ONE policies on sharing data and materials.

## Requirement 6. Reference list

The journal requested that the reference list be reviewed for completeness and accuracy and that retracted articles be handled appropriately.

**Response:** We reviewed the reference list and corrected bibliographic details where necessary. We did not identify any retracted articles or expressions of concern. We revised the references to follow the PLOS author-list format, added the published correction notices for the Furusawa et al. and Bolyen et al. articles, and updated the corresponding in-text citation numbers.

## Additional formatting revision. Supporting Information captions

In addition, we moved the Supporting Information captions to the end of the manuscript, after the References, to align with PLOS ONE manuscript organization requirements.

# Reviewer #1

## Comment 1. Sample size calculation

This manuscript is a study protocol for investigating effect of inulin supplementation on gut microbiota in patients with oral lichen planus using a double-blind, randomised, placebo-controlled parallel-group trial. Statistical analysis methods look good. But I have minor comments on the sample size calculation.
Based on the given information — two-sided significance level of 0.05, power 80%, and Cohen's d = 0.67, the sample size should have been 36 participants per group without drop out. With 10% drop out rate, the target sample size should be 40 participants per group, for a total of 80 instead of 60 participants.

**Response:** We thank the reviewer for carefully reviewing the sample size calculation and agree with the comment. We have revised the Methods section to clarify that approximately 36 participants per group are required before accounting for dropout. After allowing for an anticipated dropout rate of approximately 10%, we increased the target sample size to 40 participants per group (80 participants in total).

## Related manuscript revisions following the sample size update

**Response:** In addition to revising the sample size calculation, we updated the related descriptions in the Abstract, Randomization subsection, trial status and recruitment feasibility section, and protocol version statement to maintain consistency with the revised target sample size. The ethics amendment corresponding to the revised target sample size of 80 participants was approved by the Clinical Research Ethics Committee of Kyushu University Hospital on 16 July 2026. The UMIN registry and the ethics committee-approved protocol provided as S2 File have been updated accordingly.

We again thank the Academic Editor, journal staff, and Reviewer #1 for their helpful comments, which have improved the clarity and accuracy of the manuscript.

Sincerely,

Naoki Kaneko, on behalf of all authors
